# Temperate Intertidal Ecosystems are Functionally Richer but More Vulnerable to Loss Than Tropical Ecosystems

**DOI:** 10.1002/ece3.70657

**Published:** 2024-12-05

**Authors:** Matilda Murley, Renae K. Hovey, Jane Prince

**Affiliations:** ^1^ School of Biological Science University of Western Australia Crawley Western Australia Australia; ^2^ The UWA Oceans Institute Crawley Crawley Western Australia Australia

**Keywords:** functional bioregions, functional diversity, gastropods, invertebrate ecology, latitudinal gradient

## Abstract

Gastropods are major contributors to a range of key ecosystem services on intertidal rock platforms, supporting trophic structure in both terrestrial and marine contexts and manipulating habitat complexity. However, the functional structure of these assemblages is rarely examined across broad spatial scales. Here, we describe patterns in gastropod functional diversity, redundancy and vulnerability to functional loss across a latitudinal gradient following the west coast of Australia (18° S–34° S). Specifically, we created a trait matrix based on six categorical traits for 186 gastropod species from 39 sites to examine how trait composition varied with latitude. We found there was no latitudinal gradient in either functional richness or distinctiveness despite clear gradients in species richness and taxonomic distinctiveness, which both increased towards the equator. We delineated two distinct functional bioregions, a temperate south (34° S–27° S) and a tropical north (24° S–18° S), and found that the temperate bioregion had greater functional richness and uniqueness but lower redundancy compared to the tropical bioregion. Our findings show that gastropod assemblages in the temperate bioregion are more vulnerable to functional loss as their functional entities are supported by fewer or even single species. Comparatively, the tropical bioregion reported higher redundancies, which could provide a buffer against future change. Understanding the functional structure of intertidal ecosystems is vital as gastropods face the uncertain impacts of coastal tropicalisation, range shifts and sea level rise.

## Graphical abstract

Here, we investigated patterns in gastropod functional diversity, redundancy and vulnerability along a latitudinal gradient spanning from 18° S to 34° S along the west coast of Australia. We observed no latitudinal gradient in either functional richness or distinctiveness, despite clear gradients in species richness and taxonomic distinctiveness. However, our results indicate that temperate assemblages are more vulnerable to functional loss as their functional entities are supported by fewer or even single species.

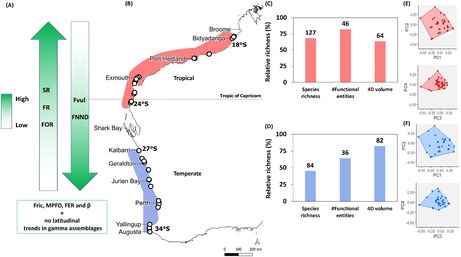

## 1 Introduction

Functional diversity is overtaking taxonomically derived species richness as the most relevant and meaningful metric to examine biodiversity in the changing world of the Anthropocene (Somerfield et al. 2008; McGill et al. 2015; Hillebrand et al. 2018). Functional diversity refers to the diversity of organism characteristics relating to interactions with their abiotic and biotic environment. It can encapsulate a range of morphological, behavioural, physiological and phenological characteristics of species that impact fitness by regulating growth, reproduction and survival (Wright et al. 2006; Violle et al. 2007; Laureto, Cianciaruso, and Samia 2015). Maintaining a diverse range of functional roles within an ecosystem ensures the stability of the system and supports the provision of ecosystem services (Cardinale et al. 2012; McWilliam et al. 2018). Functional diversity is independent of species richness and, after a certain threshold, functional richness (Fric) will stop increasing with species richness (Mouillot et al. 2014; McLean et al. 2019; Mouton et al. 2020). Changes in community composition do not necessarily induce changes in trait diversity, but if some functional roles are only performed by a few species, the loss of these species will have large impacts on overall ecosystem functioning (Violle et al. 2007; McLean et al. 2019).

When multiple species support similar or overlapping functional roles, they are said to be functionally redundant (Fonseca and Ganade 2001). A high degree of functional redundancy (FR) will help buffer against the loss of ecosystem functioning following declines in species richness, providing ecosystems with a stabilising effect and ‘reservoir of resilience’ (Mouillot et al. 2013a; McWilliam et al. 2018). Functional entities (FEs) can be used to examine FR in a system by identifying unique combinations of traits to classify species into groups of identical functions (Mouillot et al. 2014). The more species in each FE, the greater the FR of that ecosystem function (Mouillot et al. 2014). Conversely, functional roles that are not represented by several species can be considered functionally vulnerable and at risk of being lost from an ecosystem following a disturbance or decrease in species diversity (Mouillot et al. 2014).

Latitudinal diversity gradients in species richness are one of the most prevailing patterns in nature; however, it is not yet known or documented if this pattern is reflected in Fric (Schumm et al. 2019). The tropics report higher species richness and therefore higher FR than temperate regions; however, Fric can often be greater in areas with lower redundancy or higher functional uniqueness (Mouillot et al. 2014; Violle et al. 2017; Schumm et al. 2019). Examining functional diversity across species richness gradients can provide insights into the number of species supporting the same functions and how species turnover can affect functional diversity (McWilliam et al. 2018). Our understanding of functional diversity across large‐scale spatial gradients is restricted to particular regions or environments and biased towards taxa (particularly fish and corals) (Mouillot et al. 2014; McWilliam et al. 2018; Myers et al. 2021b). Documenting Fric of communities along latitudinal gradients can be useful to understand the components of biodiversity that contribute to essential functions of assemblages (McWilliam et al. 2018). Examining the components of functional diversity can also delineate distinct bioregions and identify areas that exhibit exceptionally high levels of functional diversity termed ‘functional hotspots’ (Ficetola, Mazel, and Thuiller 2017; Rumm et al. 2018; Myers et al. 2021b). Additionally, considering spatial differences in functional diversity can give powerful insights into how communities may shift and respond to future climate change or anthropogenic stresses (Mouillot et al. 2013a, 2014; Floyd et al. 2020).

Intertidal rock platform ecosystems are among some of the most productive ecosystems in the world and facilitate vital transfer of nutrients and energy (Paine 1994; Vinagre et al. 2018). Intertidal platforms support diverse invertebrate assemblages in dynamic conditions that require species to be resilient against fluctuations in wave exposure, temperature and hypoxia (Benedetti‐Cecchi 2014; Collins, Clark, and Truebano 2023; Scrosati and Ellrich 2024). Harsh conditions tend to filter organism and ecosystem functions more strongly, making intertidal ecosystems ideal for gaining insights into future climate change responses of invertebrates, as species already live close to their tolerance limits (Helmuth et al. 2006; Swenson, Anglada‐Cordero, and Barone 2011; Siefert et al. 2013). Currently, our understanding of coastal functional ecology is relatively limited for invertebrates other than corals (Przeslawski et al. 2008; McWilliam et al. 2018; Floyd et al. 2020).

Gastropods form a diverse and ecologically significant part of intertidal assemblages (Underwood 2000; Miloslavich et al. 2013). From a functional perspective, gastropods are unique because of their ubiquity and abundance, meaning they perform and contribute to a variety of ecological functions (Paine 1994; Benedetti‐Cecchi 2014). The dominance of gastropods in assemblages makes them a valued source of food for higher consumers (both terrestrial and marine), while also interacting and influencing trophic dynamics through grazing and predation (Ellis et al. 2007; Vinagre et al. 2018; Belen 2019). Gastropods help to maintain the dynamics and structure of intertidal communities through their boring and grazing behaviour, which manipulates habitat complexity and provides microhabitats for other organisms (Przeslawski et al. 2008; Belen 2019). The relatively short life spans and limited mobility of gastropods make them vulnerable to unfavourable conditions (Przeslawski et al. 2008; Beauchard et al. 2017). The lack of functional analyses of gastropods currently hinders our understanding of their functional roles in the intertidal environment (Floyd et al. 2020).

A latitudinal diversity gradient in both species' richness and taxonomic distinctness in intertidal molluscs has been identified on rock platforms along the coast of Western Australia (WA) (Murley, Hovey, and Prince 2024). Prior to this, the last comprehensive survey of coastal gastropods in WA was conducted in 1980, which defined a clear split between tropical and temperate fauna along the coastline around 28° S but did not examine functional traits of species (Wells 1980). Here, we quantify several functional traits to attempt to capture the range of ecological strategies for gastropods on 39 rock platform sites across 15° of latitude in WA. This study aims to build a spatial understanding of the functional ecology of gastropods on intertidal rock platforms. The study will investigate: (1) Does gastropod functional diversity change along a latitudinal gradient?; (2) How do FEs and trait spaces change along the latitudinal gradient?; and finally, (3) How does FR change with latitude? This approach aims to provide insight into the functioning of gastropod assemblages and form a basis for the continued collation of trait information for marine gastropods in WA.

## 2 Methods

### 2.1 Gastropod Sampling

**FIGURE 1:**
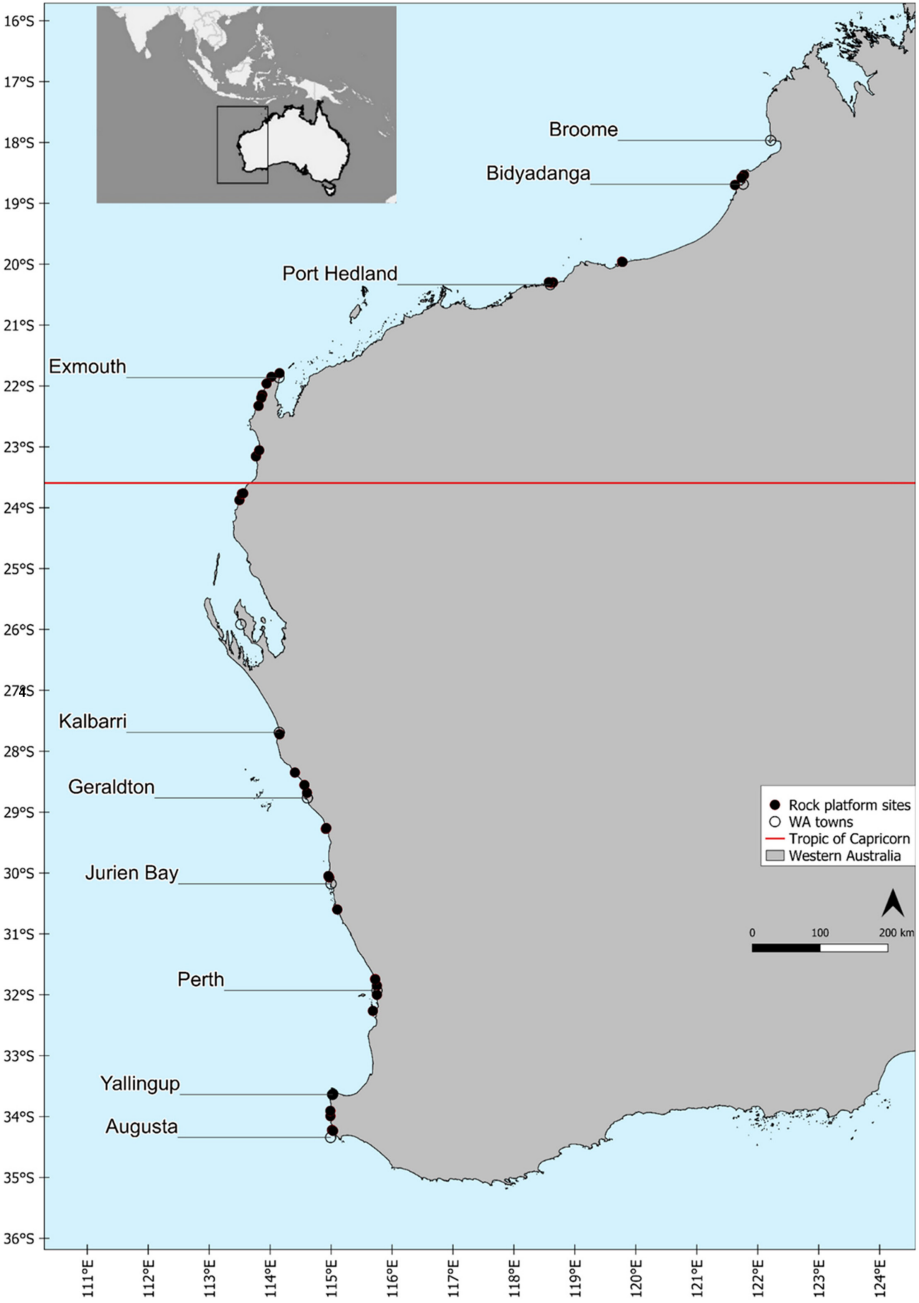
Intertidal rock platform sampling locations across the west coast of Australia with regional towns shown.

We surveyed a total of 39 limestone rock platform sites along the coast of WA in the spring/summer of 2019/2020, ranging from 18° 31′ S to 34° 14′ S (Figure 1, Table S1). Sites were sampled across 15° of latitude, excluding a break in the sampling design around Shark Bay between 26° S and 24° S. The coastline in this region is very isolated and the rock platforms identified were deemed too dangerous and inaccessible. The study endeavoured to survey three sites within each latitude; however, this was limited at some latitudes due to the absence or inaccessibility of platforms.

Gastropod species richness data were collected from rock platforms using visual surveys conducted at low tide. Platforms along the WA coast are horizontal rather than sloping. At each site, 30 1 m^2^ quadrats were stratified across the platform according to the outer, middle and inner areas of the platform, with the inner area referring to the area of the platform closest to the shore. Ten quadrats were then haphazardly placed within each stratified area and intensively searched for all gastropods above 5 mm. In addition, timed searches were conducted at each location, allocating 10 min of searching to each stratified area to record any species that were not captured by quadrats. Timed searches are useful for recording rare, cryptic or lower abundance species, making it a powerful qualitative method to use in conjunction with more traditional sampling (Paine 1994). Any species that could not be identified were collected or photographed to be examined ex situ using textbooks and online databases to classify to the lowest possible taxonomic level with certainty following the World Register of Marine Species nomenclature (Ahyong et al. 2023). If species names were unknown, open nomenclature qualifiers (e.g., sp. 1 and sp. 2) based on morphological differences were adopted to separate and organise organisms for analysis (Sigovini, Keppel, and Tagliapietra 2016).

### 2.2 Gastropod Trait Space

Trait data were collected from searches of the primary literature, specialised field guides and reputable websites (e.g., Atlas of Living Australia or World Register of Marine Species) (Ahyong et al. 2023; Atlas of Living Australia 2023). In particular, ‘Australian Marine Shells’ (Wilson 1993a, 1993b) was highly useful for gathering trait information specific to Australian species. When the data for a given species or taxon were unavailable, we used the values of the closest taxonomic relative resident in the region, following Ramírez‐Ortiz et al. (2017). Any trait for which information was available, or could be inferred, for all 189 taxa was included, resulting in no missing trait values. Traits were chosen to help identify and measure the functional roles of species and capture the key differences in trophic structure, feeding strategies, reproductive potential and dispersal methods, morphology and biomass (Myers et al. 2021b). Six categorical traits were selected based on those utilised by Floyd et al. 2020, including feeding strategy, physical position on rock platform, mobility, shape, reproductive strategy and maximum length (Table S2). These traits were chosen to balance minimising artificial functional redundancies and prevent the creation of excessive unique trait combinations (Laureto, Cianciaruso, and Samia 2015; Beauchard et al. 2017).

### 2.3 Data Analysis

#### 2.3.1 Species Richness

The gastropod assemblages of each site were pooled from both quadrat and timed search data and used to examine latitudinal trends in alpha diversity. Regional species richness was examined using gamma assemblages, which are the total species richness of the combined sites at each latitude (Whittaker 1972). Taxonomic distinctness provides insight into the phylogenetic structure of a community beyond simple species counts and measures the average taxonomic distance (e.g., genus, family and order levels) between all pairs of species, assessing the diversity within a sample based on how closely related its species are (Clarke and Warwick 1998; Clarke and Gorley 2006). To determine if species richness and average taxonomic distinctness (Δ+) exhibited latitudinal gradients, we carried out linear regression analyses using distance‐based linear models (DistLMs) to evaluate the direction and scale of any variations (Anderson, Gorley, and Clarke 2008). We also used pairwise permutational multivariate analysis of variance (PERMANOVAs), using the single fixed factor of latitude to compare diversity metrics between and within latitudes. *P* values were obtained with 9999 permutations of residuals under the reduced model. Analyses were carried out using PRIMER v7 software with the PERMANOVA+ add‐on (Clarke and Gorley 2006; Anderson, Gorley, and Clarke 2008).

#### 2.3.2 Functional Trait Diversity

Analysis of trait diversity was conducted in R studio using the R package mFD version 1.0.3 (Magneville et al. 2022; R Core Team 2022). Pairwise functional trait‐based distances between site assemblages were computed using the Gower distance as traits were not continuous and it allows variables to be equally weighted (Magneville et al. 2022). A Principal Coordinates Analysis (PCoA) was performed using this functional distance matrix and the quality of this space was assessed by examining the mean of absolute deviations (MADs) and positive eigenvalues to determine the appropriate number of PCoA axes to build the multidimensional functional space. Four dimensions were selected to represent the global hull as it captured 97% of variation in assemblages (Table S3).

Three multidimensional site‐level (alpha diversity) and latitudinal‐level (gamma diversity) functional indices were calculated to examine patterns in assemblage trait composition with latitude. Fric represents the functional space filled by an assemblage and is measured as the volume of the assemblage in proportion to the global convex hull (Villéger, Mason, and Mouillot 2008). Mean pairwise functional distance (MPFD) is the functional analogue to Δ + and is highly correlated with functional dispersion (Laliberté and Legendre 2010). MPFD is useful to determine the breadth of trait diversity in an assemblage. Functional mean nearest‐neighbour distance (FNND) measures the weighted distance to the nearest neighbor within the assemblage (Weiher et al. 1998). The FNND can be used to quantify the packing of trait space and the extent to which species occupy dissimilar niches (Magneville et al. 2022). The FNND is usually negatively correlated with species richness (Swenson and Weiser 2014). DistLMs, PERMANOVAs and graphical analysis were conducted in PRIMER to evaluate the direction and scale of any variations in all mentioned indices with latitude and species richness (Clarke and Gorley 2006, Anderson, Gorley, and Clarke 2008).

To examine turnover of functional trait composition of species between pairs of assemblages, beta diversity (β) was calculated by the mFD package using Jaccard dissimilarities and referred to as ‘functional turn over’ (Anderson et al. 2011; Swenson, Anglada‐Cordero, and Barone 2011). Jaccard dissimilarity was decomposed into ‘turnover’ and ‘nestedness’ additive components (Magneville et al. 2022). The Jaccard ‘turnover’ component is highest if there are no shared trait combinations between the assemblage pair, and the ‘nestedness’ component is highest if one assemblage hosts a small subset of the functional traits present in the other (Villéger, Grenouillet, and Brosse 2013; Magneville et al. 2022).

#### 2.3.3 FEs

FEs are created when species share the same trait values (Mouillot et al. 2014). The set of six functional traits and their respective number of categories yield a theoretical number of 4032 unique FE combinations. However, the global species pool of 186 gastropod species filled only 56 FEs (1.4%). Mouillot et al. (2014) proposed four functional indices to examine FE structure of assemblages—FE richness (FER), FR, functional over‐redundancy (FOR) and functional vulnerability (FVul). FER refers to the number of FE present in each assemblage. FR reflects the average number of species in FEs in an assemblage, whereas FOR represents the proportion of species in the FE that are above the average redundancy (Mouillot et al. 2014; Magneville et al. 2022). FV determines the proportion of FEs in an assemblage that is only represented by one species (Mouillot et al. 2014). Similarly, latitudinal and bioregional patterns in these indices were investigated in PRIMER and PERMANOVA+ using distLMs (Anderson, Gorley, and Clarke 2008).

#### 2.3.4 Functional Biogeography

Gamma assemblages using presence/absence of FEs for each latitude were generated. Cluster analysis was performed in PRIMER using a Jaccard dissimilarity matrix of the FE latitudinal assemblages using a hierarchical method with group‐average linking (GPA) to build similarity dendrograms. GPA cluster analysis was selected as it has been proven to be the most reliable at highlighting structure within the data and identifying distinct regions of diversity along the coast (Kreft and Jetz 2010). Similarity profile analysis (SIMPROF) was run for the dendrogram, using 9999 permutations, to indicate significant group structure at *p* < 0.05 (Clarke and Gorley 2006). Clustered latitudes are hereafter referred to as ‘functional bioregions’ (Woolley et al. 2019; Myers et al. 2021b). A single fixed‐factor PERMANOVA was used to compare FE composition between regions to identify any regions that could be combined (Anderson, Gorley, and Clarke 2008).

Finally, we built a functional space using the FE latitudinal assemblages and plotted regional hulls of the functional bioregions (Magneville et al. 2022). A map of cluster regions was created based on the Geocentric Datum of Australia 1994 using QGIS version 3.24.1 (QGIS Development Team 2023).

## 3 Results

### 3.1 Latitudinal Patterns in Species Richness and Functional Indices Assemblage Diversity

**FIGURE 2:**
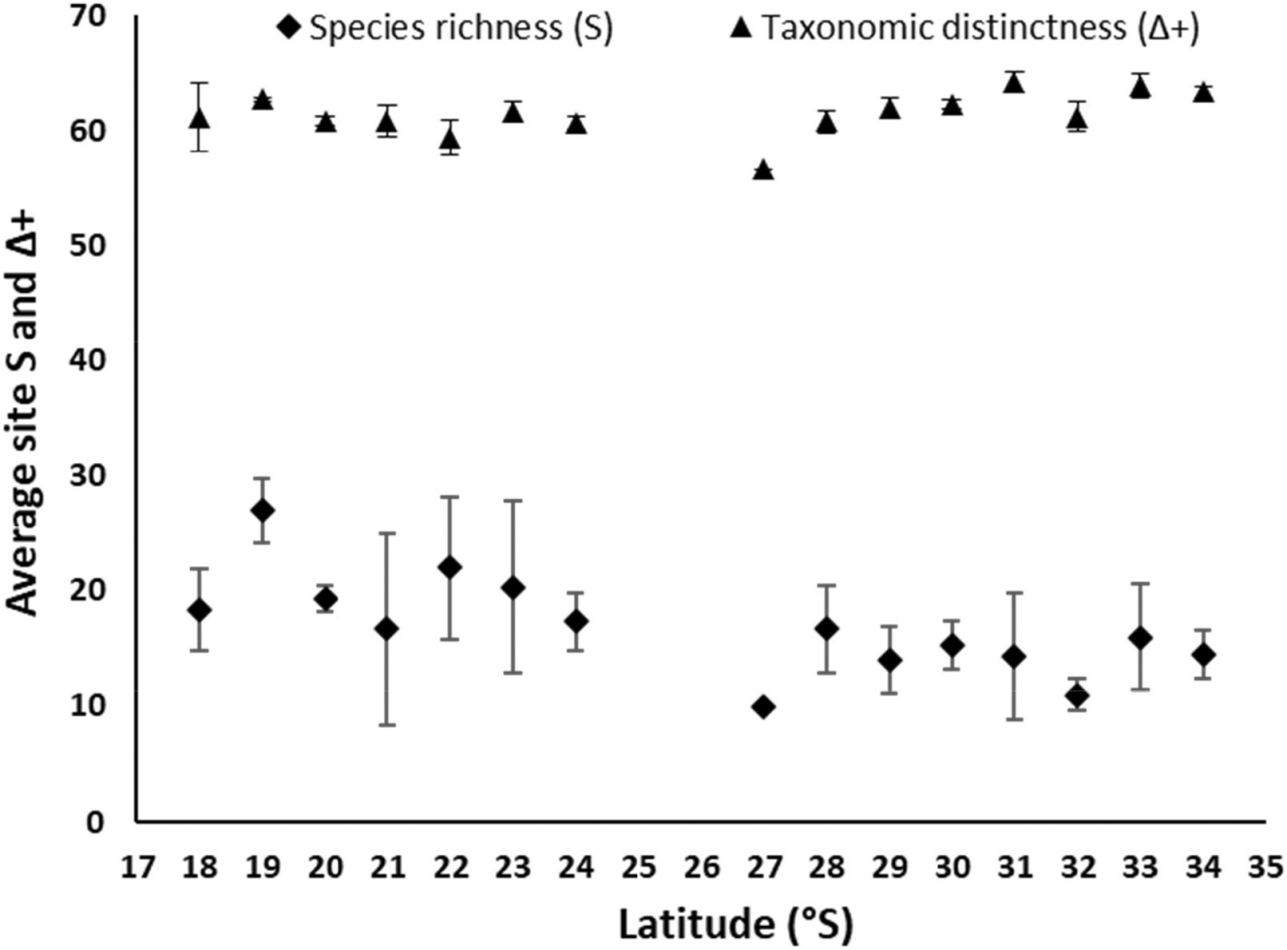
The relationship of average site species richness (α diversity, black diamonds) and site taxonomic distinctness (Δ+, black triangles) of intertidal gastropods with latitude. The number of sites sampled in each latitude varied: 34° S *n* = 2, 33° S *n* = 3, 32° S *n* = 2, 31° S *n* = 3, 30° S *n* = 3, 29° S *n* = 2, 28° S *n* = 3, 27° S *n* = 1, 24° S *n* = 3, 23° S *n* = 3, 22° S *n* = 3, 21° S *n* = 3, 20° S *n* = 3, 19° S *n* = 2, and 18° S *n* = 3.

This study recorded the abundance, distribution and trait information of 186 species of gastropods from 63 families over 15° latitude along the coast of WA. Site taxa richness increased towards the equator (DistLM, Pseudo *F*(1,37) = 11.40, *p* = 0.0021), as did average site richness (alpha diversity (*α*)) for each latitude, with latitude explaining 48.50% of the variation in taxa richness (DistLM, Pseudo *F*(1,13) = 12.23, *p* = 0.0028) (Figure 2). Combined latitudinal species richness (gamma diversity (γ)) displayed a similar gradient along the coastline, with latitude explaining 49% of variation (DistLM, (*F*(1,13) = 12.61, *p* = 0.004)). This highlights that across site and regional scales, species diversity increased towards lower latitudes. Site taxonomic distinctness (Δ+) exhibited an inverse trend to species richness, with Δ+ increasing towards higher latitudes (Figure 2). Site Δ+ significantly increased towards higher latitudes with latitude explaining 20.22% of variation (DistLM, *F*(1,37) = 9.38, *p* = 0.0037). However, this was not seen at a regional scale, as when total Δ+ (γ) was regressed against latitude, there was no significant gradient (DistLM, (*F*(1,13) = 0.70, *p* = 0.45); Figure 2).

### 3.2 Functional Indices

**FIGURE 3:**
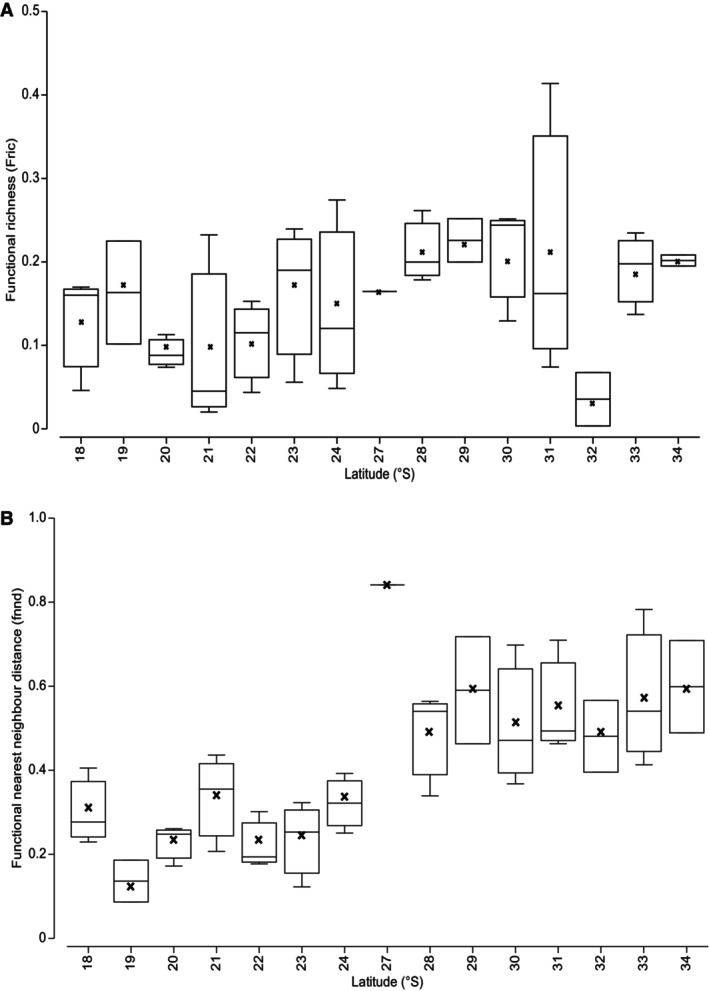
(A) Functional richness (Fric) and (B) Functional mean nearest neighbour distance (FNND) of Western Australian intertidal gastropod assemblages across sites along a latitudinal gradient. The median is indicated by the horizontal line within each box. The mean is indicated by the black cross within each box. The number of sites sampled in each latitude varies: 34° S *n* = 2, 33° S *n* = 3, 32° S *n* = 2, 31° S *n* = 3, 30° S *n* = 3, 29° S *n* = 2, 28° S *n* = 3, 27° S *n* = 1, 24° S *n* = 3, 23° S *n* = 3, 22° S *n* = 3, 21° S *n* = 3, 20° S *n* = 3, 19° S *n* = 2 and 18° S *n* = 3.

Fric remained relatively similar across all assemblages along the coastline (Figure 3A). At the site level, latitude only explained 10% of variation in Fric between assemblages but was still significant (DistLM, (*F*(1,37) = 4.28, *p* = 0.045)). When Fric of gamma assemblages was regressed against latitude, there was no significant relationship (DistLM, (*F*(1,13) = 0.21, *p* = 0.65); Figure S1) but functional mean pairwise distance (MPFD) did significantly vary with latitude at the site level (DistLM (*F*(1,37) = 5.92, *p* = 0.021; Figure S2)), although latitude only explained 13.8% of MPFD variation (Figures S1 and S2). MPFD of gamma assemblages was not significantly explained by latitude (DistLM, (*F*(1,13) = 1.10, *p* = 0.31); Figure S3). When site‐level functional mean nearest neighbour distance (FNND) was regressed against latitude, FNND significantly increased towards higher latitudes, with latitude explaining 53% of variation (DistLM (*F*(1,37) = 41.63, *p* = 0.0001; Figure 3B)). The FNND of gamma assemblages was also significantly explained by latitude (DistLM (Pseudo *F*(1,13) = 8.88, *p* = 0.013); Figure S4).

#### 3.2.1 Functional Turnover

Functional β diversity was relatively high within latitudes, with a mean of 65.24% ± 18.80% average Jaccard dissimilarity. There was no relationship between latitudinal turnover and latitude, indicating that β diversity within latitudes was similar across all latitudes (Figure S5). The average Jaccard dissimilarity between latitude turnover was 55.33% ± 17.9%, with no relationship between turnover and distance between latitudes (Figure S6). Kalbarri (27° S) exhibited high turnover with all other latitudes, and when Jaccard dissimilarity was decomposed, it was found to be dominated by nestedness (Table S4). This is reflective of how the Kalbarri species assemblage represented a small subset of the functional traits at other latitudes due to the low number of species (*n* = 9). At all other latitudes, locations that were further apart geographically had Jaccard dissimilarity components that were dominated by turnover of species assemblage trait composition (Table S4). This demonstrates that most of turnover along the coast was driven by the lack of shared trait combinations between the assemblage pairs.

#### 3.2.2 FEs

Analysis of the trait space grouped the species assemblage into 56 FEs. When site assemblages were composed of FEs instead of species, the number of FEs did not significantly differ between latitudes ((*F*(14,24) = 0.68, *p* = 0.77), Figure S7). Species richness explained 76.4% of variation in the number of FEs, with the number of FEs increasing at sites with higher species richness (DistLM, (*F*(1,37) = 120, *p* = 0.0001)). However, this relationship appears to be approaching the threshold where FER will stop increasing with species richness (Figure S8). FR of site assemblages significantly increased towards lower latitudes (DistLM, (*F*(1,37) = 34.13, *p* = 0.0001), Figure S9). Similarly, FOR in site assemblages also significantly increased towards lower latitudes (DistLM, (*F*(1,37) = 42.11, *p* = 0.0001), Figure S10). It follows that the FVul of site assemblages significantly increased towards higher latitudes, with latitude explaining 48.5% of variation (DistLM (*F*(1,37) = 34.79, *p* = 0.0001), Figure S11).

### 3.3 Functional Biogeography

**FIGURE 4:**
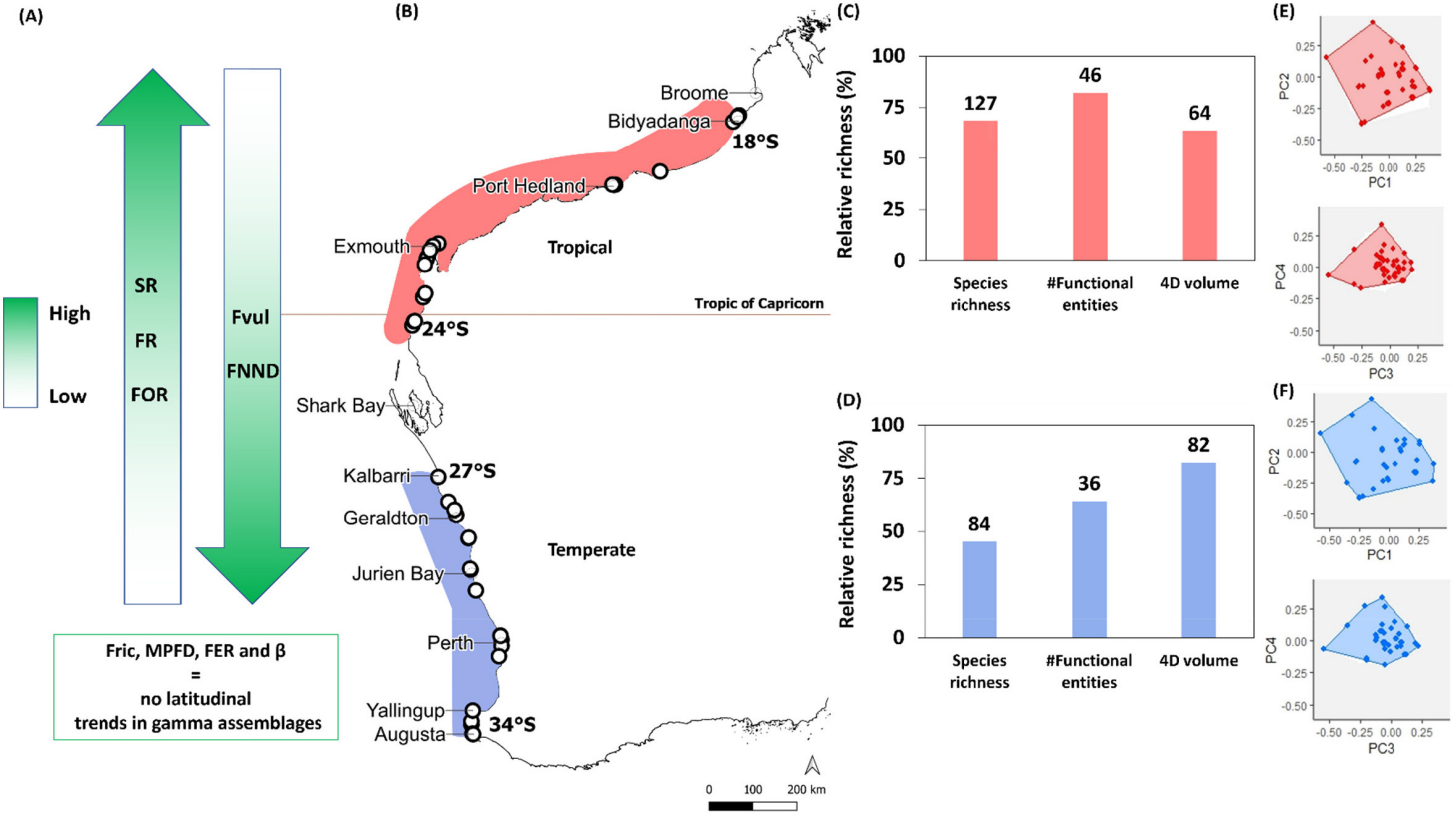
(A) Summary of the direction of latitudinal trends in site assemblages for various diversity indices (FR, functional redundancy; FOR, functional over redundancy; Fvul, Functional vulnerability; Fric, Functional richness; MPFD, functional mean pairwise distance; #FEs, number of FEs; SR, species richness; β, beta diversity; Δ+, average taxonomic distinctness) (B) Functional bioregions of intertidal gastropod assemblages in Western Australia. The tropical bioregion spans 18° S–24° S shown in red and the temperate bioregion spans 27° S–34° S shown in blue. Sampling sites are indicated by white circles. Histograms C (tropical) and D (temperate) show species richness, FER (number of unique trait combinations) and functional richness (trait space volume filled by each assemblage), expressed as a percentage relative to the global pool. Number of species and number of FEs are displayed above corresponding bars. Distributions of FEs are shown in functional spaces (E (tropical) and F (temperate)) where axes represent PC1–PC2 and PC3–PC4 from a principle coordinate analysis of functional traits. The global convex hull, including 186 species split into 56 FEs, is shown in white. The colour‐filled areas show the functional volume filled by each assemblage. Coloured circles represent the FEs present in the fauna, filled points represent FEs present in the fauna that are the vertices of the convex hull (e.g., the ones that shape edges of coloured area) and grey crosses represent FEs absent in the fauna.

To further investigate any broad‐scale patterns in functional biogeography along the coast, GPA cluster analysis was conducted using the latitudinal FE assemblages. Hierarchical cluster analysis based on Jaccard dissimilarities revealed that latitudes could be separated into three statistically significant distinct groups (*k* = 3) (Figure S12). Differences among cluster groups were further tested using pairwise PERMANOVA and showed significant differences in FE assemblage composition between temperate and tropical (*t* = 2.6559, *p* = 0.0001), Kalbarri and tropical (*t* = 1.3303 *p* = 0.0453), but not temperate and Kalbarri clusters (*t* = 1.0153, *p* = 0.5273; Table S4). Following this, Kalbarri was combined with the temperate bioregion. This enabled the WA coastline to be split into two distinct biogeographic regions, the temperate south (34° S–27° S) and a tropical north (24° S—18° S; Figure 4).

Functional turnover (β diversity) of the FE assemblages between the two bioregions was moderately high (Table 1). When the Jaccard dissimilarity was decomposed into turnover and nested components, it was found to be dominated by turnover, indicating that trait composition replacement was driving the differences in FE assemblage in each bioregion.

**TABLE 1:** Components of intertidal gastropod functional turnover (β diversity) based on Jaccard dissimilarities between functional bioregions in Western Australia.

Bioregion		Jaccard dissimilarity	Jaccard turnover	Jaccard nestedness
Temperate	Tropical	0.4381718	0.296	0.142172

A principal component analysis (PCA) of the global trait space based on the FE latitudinal assemblages revealed that there were four significant axes of correlated trait variation with 70% of variation expressed in just two dimensions and 97% in four dimensions (Figure S13). The tropical hull occupied 64% of the global hull and the temperate hull occupied 82% (Figure 4). The temperate region occupied the most trait space despite having less FEs than the tropical region. This indicates that the traits represented by the temperate taxa were rarer or expressed more extreme trait values than the tropical fauna by dispersing more widely between the global hull boundaries.

#### 3.3.1 FEs of Bioregions

**FIGURE 5:**
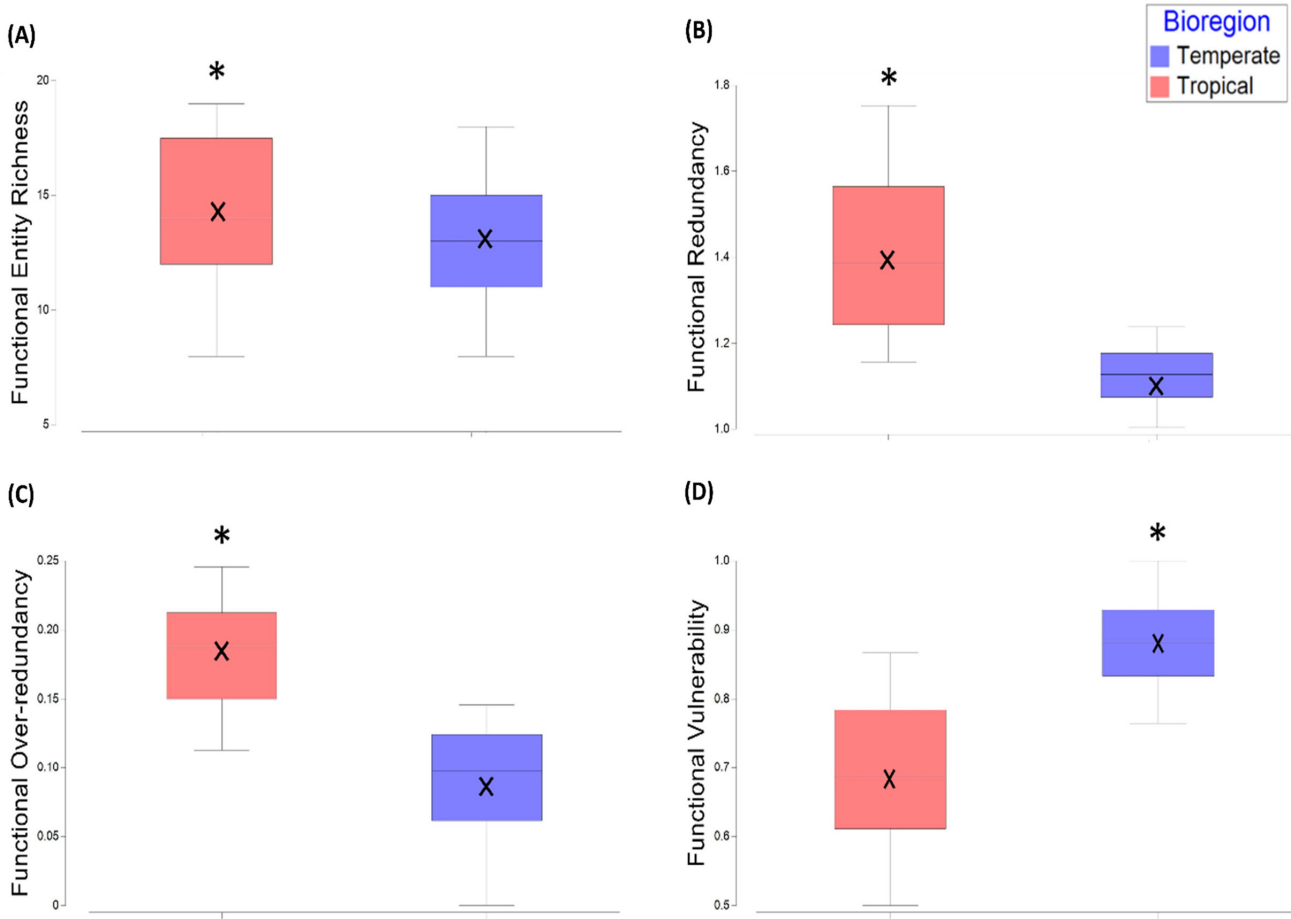
(A) Functional entity richness, (B) functional redundancy (Fred), (C) functional over‐redundancy (FOR) and (D) functional vulnerability (Fvul) of intertidal gastropod assemblages in temperate (blue, *n* = 19 sites) and tropical (red, *n* = 20 sites) functional bioregions. The median is indicated by the horizontal line within each box. The mean is indicated by the black cross within each box. Significance is indicated by an asterisk.

The number of FEs did not significantly differ between temperate and tropical bioregions (Figure 5A). However, FR of FEs was significantly higher in the tropical bioregion (*F*(1,37) = 39.44, *p* = 0.0001), indicating the number of species in each FE in tropical assemblages was greater than FEs in temperate assemblages (Figure 5B). FOR was also significantly higher in the tropical bioregion (*F*(1,37) = 44.82, *p* = 0.0001). There was a relatively low average FOR across both bioregions, indicating that both assemblages had relatively even numbers of species within FEs (Figure 5C). This means that up to 86% of temperate species and 76% of tropical species did not fill FEs above the mean level of FR. FVul was significantly higher for the temperate bioregion (*F*(1,37) = 39.605, *p* = 0.0001) with up to 100% of FEs in a given site assemblage composed of a single species (Figure 5D). The average FVul for the tropical bioregion was 69%, indicating that a high proportion of FEs in these assemblages were also only represented by a single species.

## 4 Discussion

Our results show that the richness and breadth of gastropod functional diversity in intertidal assemblages do not significantly change along the WA coastline. The absence of a latitudinal gradient in Fric and distinctness (MPFD) demonstrates that the richness and breadth of functional trait diversity are maintained along the coastline. Although a species richness gradient was documented, with richness increasing towards lower latitudes, there was a decoupling between taxonomic and functional diversity. The increasingly greater breadth of average taxonomic distinctness (Δ+) at temperate latitudes could explain how functional diversity was able to be maintained across the thermal and latitudinal gradient along the WA coast (Miranda et al. 2005). Unlike latitudinal gradients in species richness, spatial patterns in functional diversity are less well known globally with some studies reporting functional gradients with latitude (Ramírez‐Ortiz et al. 2017; Schumm et al. 2019; Thyrring and Peck 2021), whereas others found stronger functional gradients associated with other factors like depth or nutrient availability (Zintzen et al. 2017; Rojas‐Montiel et al. 2020).

Functional turnover was relatively high and did not vary significantly within or between latitudes or show any latitudinal trends. Functional turnover was driven by species replacement between the assemblages rather than changes in the numbers of species between sites. Despite this variation, some high‐level filtering processes must be occurring to form bioregions with distinct FE assemblage compositions. The ‘functional village hypothesis’ developed by Myers et al. (2021b), adapted from Smith et al. (2014), considers that sites with similar environmental characteristics will support certain ‘essential functional components’, creating functionally similar ecosystems. This phenomenon has been found in other ecosystems, where environmental filtering has created a convergence of certain traits (Swenson, Anglada‐Cordero, and Barone 2011; Siefert et al. 2013; Pinto‐Ledezma, Larkin, and Cavender‐Bares 2018). As limestone rock platforms in WA exhibit relatively homogeneous physical habitat this could allow for environmental filtering of traits and favour certain niches of function (Smith et al. 2014; Myers et al. 2021b). The number of FEs does not vary between tropical and temperate bioregions, but the FE assemblage composition does, suggesting that there could be a unique ‘functional village’ for each. Fluctuations in Leeuwin current strength, larval supply and thermal gradients could be possible drivers of this apparent functional filtering, but this remains to be investigated (Wilson and Kirkendale 2016; Caputi et al. 1996).

Functional indices and entities allow for further insights into the community trait structure of temperate and tropical bioregions. The increase of delta + in sites towards higher latitudes could have also bolstered the functional mean nearest neighbour distance (FNND) of temperate ecosystems to create more disparate trait assemblages, as species at higher latitudes occupied more dissimilar niches than at lower latitudes (Mouillot et al. 2013a; Myers et al. 2021a). Functional turnover between bioregions was driven by differences in FE assemblages rather than the number of FEs present, further highlighting how temperate and tropical bioregions support distinct, unique traits (Siefert et al. 2013; Magneville et al. 2022). Not only were assemblages of the temperate bioregion more functionally disparate but they also supported a greater Fric despite having a lower species richness than the tropical bioregion. The lower species richness of temperate assemblages demonstrates how lower diversity assemblages can support high Fric (Bellwood et al. 2004; Mouillot et al. 2014). Low species diversity has been associated with high Fric in other invertebrate assemblages (Ramírez‐Ortiz et al. 2017). Studies of temperate ecosystems reporting higher than expected Fric suggest temperate environments can offer habitat and resources to suit a broader range of ecological requirements, permitting a higher degree of niche differentiation and lower competition (Lamanna et al. 2014; Rojas‐Montiel et al. 2020). Less niche packing of FEs in temperate assemblages compared to tropical has also been suggested to allow for a wider range of trait combinations to arise (Lamanna et al. 2014).

Analysis of assemblages between bioregions highlighted how FR was higher at lower latitudes. Increased FR in tropical ecosystems has been documented previously, with the greater availability of resources allowing for multiple species to occupy the same niche (Walker 1995; Martins et al. 2012; Biggs et al. 2020). High FR can help ensure the functional diversity of tropical systems to some extent by providing a buffer against species richness loss (Hidasi‐Neto, Barlow, and Cianciaruso 2012; Gallagher, Hughes, and Leishman 2013). However, the resilience of tropical assemblages to disturbances may be reduced as FOR is relatively low (up to 24%), highlighting how higher FR may not sufficiently protect against the loss of function as species are disproportionately packed into few FEs (Mouillot et al. 2014; McWilliam et al. 2018; Floyd et al. 2020). Further research investigating the distributions of individual abundances between FEs is required to identify tropical functions that are most vulnerable (Stuart‐Smith et al. 2013). Ultimately, despite being more functionally rich, the temperate bioregion was more vulnerable to functional loss. A majority of FEs within temperate assemblages were composed of a single species, which means that even the loss of a few species will reduce the breadth of functions supported (Oliver et al. 2015). Similar to our findings, other studies have shown that functionally unique species are more common in temperate rather than tropical communities (Stuart‐Smith et al. 2013). As long as key species are conserved, limited FR is not always a threat as some ecosystems have reported the loss of 75% of species richness before any functional groups disappeared (Bellwood, Hoey, and Choat 2003; Fonseca and Ganade 2001). Quantifying and identifying key functional species in marine ecosystems lags behind terrestrial research, but has begun for some communities (e.g., coral reefs and deep sea) (Bellwood et al. 2019; McWilliam et al. 2020; Myers et al. 2021a). Given the dynamic nature of the intertidal environment, more research into the effects of temporal and spatial variation on functional processes is required to further investigate the vulnerability of temperate assemblages (Zintzen et al. 2011). Especially through the lens of global climate change, it is vital to identify species that are both functionally important and also resilient to future environmental changes (Hillebrand et al. 2018).

The clear biogeographic split in functional composition illustrated by our results provides a baseline to measure species redistributions along the WA coast and impetus to further investigate the mechanisms underpinning temperate ecosystem functioning. However, the boundaries of the functional bioregions defined here are subject to shift as environmental change continues to influence communities, so increased understanding of temporal turnover between regions is required (Pinto‐Ledezma, Larkin, and Cavender‐Bares 2018; McLean et al. 2019; Myers et al. 2021b).

Globally, temperate ecosystems are under threat from habitat loss and species redistribution due to the accelerating impacts of climate change (Antão et al. 2020; Worm, Lotze, and Letcher 2021). The Indian Ocean is one of the fastest‐warming oceans in the world and the impacts of this have already begun to influence species distributions along the WA coastline (Wernberg et al. 2016; Tuckett et al. 2017; Vergés et al. 2019; Roxy et al. 2020). While posing a threat to species distributions, the tropicalisation of the WA coastline and the poleward shift of marine life due to climate change could also facilitate the opportunity for functional diversity to be maintained in temperate regions (Madin et al. 2012). Species richness is predicted to increase in the temperate regions as tropical species expand their geographic range with ocean warming (Bates et al. 2014; Worm, Lotze, and Letcher 2021). The influx of tropical climate immigrant species could increase the FR of temperate regions, allowing tropical ecosystems to act as a reservoir of functional diversity (Vergés et al. 2019; Miller et al. 2023). Although the mechanisms remain to be unravelled, the spread of tropical gastropod species into temperate ecosystems could allow for tropical species to fill any locally extinct functional niches that were affected by the loss of temperate species due to thermal intolerances (Marzloff et al. 2018; Miller et al. 2023). The recruitment of a tropical counterpart would allow for rock platform ecosystems to maintain productivity, with different species performing the existing ecological roles (Vergés et al. 2019; Kingsbury et al. 2020). Temperate niche tropicalisation has been investigated in fish and it has been shown that tropical species can perform the same functional roles as their respective temperate counterparts (Miller et al. 2023). It is difficult to predict how assemblages will respond to the simultaneous and potentially irreversible distribution shifts in the future, but there is evidence in fish that tropical and temperate species can co‐exist, while thermal tolerances permit (Zintzen et al. 2011; Kingsbury et al. 2020; Miller et al. 2023).

Sea level rise (SLR) is another aspect of climate change that will impact intertidal assemblages, where previously intertidal habitats become permanently subtidal (Kaplanis et al. 2020; Rullens et al. 2022). The south‐west coast of Australia is experiencing faster than the global average SLR and is already classified as a microtidal region, making rock platforms along this coastline particularly vulnerable to future inundation (Lowe, Cuttler, and Hansen 2021; Kaplanis et al. 2020). A study of rocky shores in Scotland found that SLR between 0.3 m and 1.9 m could result in a 10%–50% loss of intertidal extent (Jackson and Mcilvenny 2011). The change in abiotic conditions (increased periods of submersion, steeper platform slope, decreased habitat availability, etc.) that would occur with SLR would adversely impact functional groups that could not re‐establish themselves at an appropriate littoral height or on the substrate of the new intertidal zone (Thorner, Kumar, and Smith 2014; Schaefer et al. 2020). This loss of the intertidal functional niche on rock platforms will favour the recruitment of more generalist species and possibly allow the invasion of subtidal species, which were previously constrained by the dynamic tidal environmental conditions, increasing the competition for intertidal species (Schaefer et al. 2020; Rullens et al. 2022). Other studies have found that trophic generalists were more plastic in adapting to the current impacts of climate change; however, this has not been investigated in gastropods (Kingsbury et al. 2020; Monaco et al. 2020). It would be interesting to compare subtidal and intertidal assemblages to determine what traits are favoured by the intertidal environment to our knowledge this has not been addressed in marine assemblages. Increased understanding of the role environmental filtering plays in structuring ecological shifts, and habitat migrations is also required to gain insights into SLR impacts on intertidal communities.

Functional diversity can be lost at greater rates when ecosystems with low redundancy are exposed to congruent impacts (Worm et al. 2006; Mouillot et al. 2013a). In the context of WA, the south‐west region is the most densely populated, which exposes the marine environment to greater human influence and synergistic stressors (e.g., coastal development, pollution, and fishing pressure) (Mora et al. 2011; Centre for Population 2022). This intrinsically makes the temperate bioregion more vulnerable to functional loss, not only due to the structure but also from the greater exposure to anthropogenic impacts and habitat modification (Martins et al. 2012). The loss of functional diversity for temperate ecosystems is more likely in the event of environmental change or disturbance, as the multiplication of these stressors can further erode resilience, placing already vulnerable functions under additional pressure (Worm et al. 2006; Mora et al. 2011). The long‐term provisioning of ecosystem services in temperate intertidal environments will depend on the scale of the deleterious effects caused by greater population density in combination with the rate of climate change impacts (Leitão et al. 2016). Further work to identify particularly vulnerable temperate functional groups will highlight any irreplaceable species or groups that support overall intertidal function and help boost ecosystem resilience by preserving a broad range of trait diversities (Micheli and Halpern 2005).

Our results are an important first step to better understanding the functional structures and vulnerabilities of gastropod assemblages in WA. The use of additional continuous data traits would help to increase the relevancy of our results, which exclusively rely on categorical traits, however, as Floyd et al. (2020) noted the availability of mollusc traits is limited and currently there is not enough knowledge of trait plasticity in molluscs to accommodate more accurate trait coding (Fiorentino et al. 2017). Functional diversity patterns depend strongly on the traits measured and thus are susceptible to change if additional or more specific traits are included (Beauchard et al. 2017; Floyd et al. 2020). Data availability is a major restrictive factor in any functional trait analysis as phyla that receive less attention due to lack of commercial applicability or research bias are disadvantaged (Hughes et al. 2021). Recent studies of vertebrates have measured continuous traits from individuals to examine the effects of both interspecific and intraspecific variation on assemblage structure and dynamics, representing the newest iteration of functional research (Myers et al. 2021a; Diamond and Roy 2023). Gathering intraspecific information for gastropod assemblages would aid in interpreting the relative functional importance of environmental filtering and internal biotic interactions across latitudinal gradients (Myers et al. 2021a). Additionally, consideration of the correlation between traits and environmental conditions could provide further insights into how intertidal Fric varies spatially and aligns with abiotic factors (e.g., temperature, tidal regime, and wave exposure; Helmuth et al. 2006; Pinto‐Ledezma, Larkin, and Cavender‐Bares 2018). Understanding the influence of abiotic factors on trait assemblages is especially relevant given that WA has already reported evidence of functional change due to tropicalisation in subtidal environments (Bosch et al. 2022; Sahin et al. 2024). Focusing future efforts to document traits and other variables that can capture responses to climate change (e.g., size, growth rate or abundance data) will increase the scope of researchers to make future predictions of gastropod assemblages in WA (McLean et al. 2019).

## 5 Conclusion

Integrating functional trait‐based and the documentation of empirical evidence to understand Fric is becoming essential to gain increased insights into the mechanisms structuring assemblages and make conservation efforts more effective (Myers et al. 2021b; Gallagher, Hughes, and Leishman 2013). The increasing decline in species diversity globally will undoubtedly result in assemblages becoming simplified, which could lead to substantial losses in function and the disruption of ecosystem services (Fonseca and Ganade 2001). Our study provides new insights into gastropod functional diversity across a vast latitudinal gradient, characterising how the structure of intertidal communities varied according to key ecological traits ranging from food acquisition to locomotion. The absence of a Fric gradient and low latitudinal turnover support the idea of a ‘functional village’, positing that key biological functions remain similar on rock platforms across large spatial scales (Myers et al. 2021a). Gastropod FE assemblages exhibit distinct biogeographic regionalisation, which highlights the vulnerability of the more unique and rich temperate assemblages. Tropical assemblages show greater FR providing a buffer against future change and potentially acting as a reservoir of essential functions for temperate assemblages as coastline tropicalisation continues (Mouillot et al. 2013b; Vergés et al. 2019). Already facing the uncertain impacts of range shifts and SLR, temperate intertidal assemblages are potentially at more risk from synergistic anthropogenic impacts threatening functional diversity as well (Antão et al. 2020; Kaplanis et al. 2020). This study establishes important baselines for gastropod assemblages against which future changes may be compared, allowing hypotheses regarding the potential mechanisms to be refined and management decisions to be better informed. Ultimately, the future of preserving intertidal assemblages relies on furthering our comprehension of traits among species and identifying FVul across regions.

## Author Contributions

**Matilda Murley:** conceptualization (lead), data curation (lead), formal analysis (lead), funding acquisition (lead), writing – original draft (lead). **Renae K. Hovey:** formal analysis (supporting), methodology (equal), resources (supporting), supervision (supporting), writing – review and editing (equal). **Jane Prince:** conceptualization (supporting), formal analysis (supporting), methodology (equal), resources (supporting), supervision (lead), writing – review and editing (equal).

## Conflicts of Interest

The authors declare no conflicts of interest.

## Supporting information


Data S1.

## Data Availability

Data have been uploaded and are available on Dryad at this URL: doi: 10.5061/dryad.3xsj3txqn. Available for reviewer access: https://datadryad.org/stash/share/tkorTfK2v‐kiJXDXpRn7ZFb‐e9zKxNH4PnGgHMPuNos.

## References

[ece370657-bib-0001] Ahyong, S. , C. B. Boyko , N. Bailly , et al. 2023. World Register of Marine Species (WoRMS).

[ece370657-bib-0002] Anderson, M. J. , T. O. Crist , J. M. Chase , et al. 2011. “Navigating the Multiple Meanings of β Diversity: A Roadmap for the Practicing Ecologist.” Ecology Letters 14: 19–28. 21070562 10.1111/j.1461-0248.2010.01552.x

[ece370657-bib-0003] Anderson, M. J. , R. Gorley , and K. Clarke . 2008. PERMANOVA+ for PRIMER: Guide to Software and Statistical Methods. Plymouth, UK: PRIMER‐E Ltd.

[ece370657-bib-0004] Antão, L. H. , A. E. Bates , S. A. Blowes , et al. 2020. “Temperature‐Related Biodiversity Change Across Temperate Marine and Terrestrial Systems.” Nature Ecology & Evolution 4: 927–933. 32367031 10.1038/s41559-020-1185-7

[ece370657-bib-0005] Atlas of Living Australia . 2023. “Atlas of Living Australia Website.” http://www.ala.org.au.

[ece370657-bib-0006] Bates, A. E. , G. T. Pecl , S. Frusher , et al. 2014. “Defining and Observing Stages of Climate‐Mediated Range Shifts in Marine Systems.” Global Environmental Change 26: 27–38.

[ece370657-bib-0007] Beauchard, O. , H. Veríssimo , A. M. Queirós , and P. M. J. Herman . 2017. “The Use of Multiple Biological Traits in Marine Community Ecology and Its Potential in Ecological Indicator Development.” Ecological Indicators 76: 81–96.

[ece370657-bib-0008] Belen, T. L. 2019. Assemblage of Gastropods in the Rocky Intertidal Zone of Asry Beach, Kingdom of Bahrain. Invertebrates. Rijeka: IntechOpen.

[ece370657-bib-0009] Bellwood, D. R. , A. S. Hoey , and J. H. Choat . 2003. “Limited Functional Redundancy in High Diversity Systems: Resilience and Ecosystem Function on Coral Reefs.” Ecology Letters 6: 281–285.

[ece370657-bib-0010] Bellwood, D. R. , T. P. Hughes , C. Folke , and M. Nyström . 2004. “Confronting the Coral Reef Crisis.” Nature 429: 827–833. 15215854 10.1038/nature02691

[ece370657-bib-0011] Bellwood, D. R. , M. S. Pratchett , T. H. Morrison , et al. 2019. “Coral Reef Conservation in the Anthropocene: Confronting Spatial Mismatches and Prioritizing Functions.” Biological Conservation 236: 604–615.

[ece370657-bib-0012] Benedetti‐Cecchi, L. 2014. “Intertidal Rocky Shores.” In Marine Community Ecology and Conservation, edited by M. Bertness , J. Bruno , B. Silliman , and J. Stachowicz . United Kingdom: Sinauer Associates INC.

[ece370657-bib-0013] Biggs, C. R. , L. A. Yeager , D. G. Bolser , et al. 2020. “Does Functional Redundancy Affect Ecological Stability and Resilience? A Review and Meta‐Analysis.” Ecosphere 11: e03184.

[ece370657-bib-0014] Bosch, N. E. , M. Mclean , S. Zarco‐Perello , et al. 2022. “Persistent Thermally Driven Shift in the Functional Trait Structure of Herbivorous Fishes: Evidence of Top‐Down Control on the Rebound Potential of Temperate Seaweed Forests?” Global Change Biology 28: 2296–2311. 34981602 10.1111/gcb.16070

[ece370657-bib-0015] Caputi, N. , W. J. Fletcher , A. Pearce , and C. F. Chubb . 1996. “Effect of the Leeuwin Current on the Recruitment of Fish and Invertebrates Along the Western Australian Coast.” Marine and Freshwater Research 47: 147–155.

[ece370657-bib-0016] Cardinale, B. J. , J. E. Duffy , A. Gonzalez , et al. 2012. “Biodiversity Loss and Its Impact on Humanity.” Nature 486: 59–67. 22678280 10.1038/nature11148

[ece370657-bib-0017] Centre for Population . 2022. 2022 Population Statement. Canberra, Australia: Australian Government.

[ece370657-bib-0018] Clarke, K. , and R. Gorley . 2006. PRIMER v7: User Manual/Tutorial. Plymouth, Uk: PRIMER‐E Ltd.

[ece370657-bib-0019] Clarke, K. R. , and R. M. Warwick . 1998. “A Taxonomic Distinctness Index and Its Statistical Properties.” Journal of Applied Ecology 35: 523–531.

[ece370657-bib-0020] Collins, M. , M. S. Clark , and M. Truebano . 2023. “The Environmental Cellular Stress Response: The Intertidal as a Multistressor Model.” Cell Stress and Chaperones 28: 467–475. 37129699 10.1007/s12192-023-01348-7 PMC10469114

[ece370657-bib-0021] Diamond, J. , and D. Roy . 2023. “Patterns of Functional Diversity Along Latitudinal Gradients of Species Richness in Eleven Fish Families.” Global Ecology and Biogeography 32: 450–465.

[ece370657-bib-0022] Ellis, J. C. , M. J. Shulman , M. Wood , J. D. Witman , and S. Lozyniak . 2007. “Regulation of Intertidal Food Webs by Avian Predators on New England Rocky Shores.” Ecology 88: 853–863. 17536702 10.1890/06-0593

[ece370657-bib-0023] Ficetola, G. F. , F. Mazel , and W. Thuiller . 2017. “Global Determinants of Zoogeographical Boundaries.” Nature Ecology & Evolution 1: 89. 28812660 10.1038/s41559-017-0089

[ece370657-bib-0024] Fiorentino, D. , R. Pesch , C.‐P. Guenther , et al. 2017. “A ‘Fuzzy Clustering’ Approach to Conceptual Confusion: How to Classify Natural Ecological Associations.” Marine Ecology. Progress Series (Halstenbek) 584: 17–30.

[ece370657-bib-0025] Floyd, M. , M. Mizuyama , M. Obuchi , et al. 2020. “Functional Diversity of Reef Molluscs Along a Tropical‐To‐Temperate Gradient.” Coral Reefs 39: 1361–1376.

[ece370657-bib-0026] Fonseca, C. R. , and G. Ganade . 2001. “Species Functional Redundancy, Random Extinctions and the Stability of Ecosystems.” Journal of Ecology 89: 118–125.

[ece370657-bib-0027] Gallagher, R. V. , L. Hughes , and M. R. Leishman . 2013. “Species Loss and Gain in Communities Under Future Climate Change: Consequences for Functional Diversity.” Ecography 36: 531–540.

[ece370657-bib-0028] Helmuth, B. , N. Mieszkowska , P. Moore , and S. J. Hawkins . 2006. “Living on the Edge of Two Changing Worlds: Forecasting the Responses of Rocky Intertidal Ecosystems to Climate Change.” Annual Review of Ecology, Evolution, and Systematics 37: 373–404.

[ece370657-bib-0029] Hidasi‐Neto, J. , J. Barlow , and M. V. Cianciaruso . 2012. “Bird Functional Diversity and Wildfires in the Amazon: The Role of Forest Structure: Bird Functional Diversity and Wildfires in Amazon.” Animal Conservation 15: 407–415.

[ece370657-bib-0030] Hillebrand, H. , B. Blasius , E. T. Borer , et al. 2018. “Biodiversity Change Is Uncoupled From Species Richness Trends: Consequences for Conservation and Monitoring.” Journal of Applied Ecology 55: 169–184.

[ece370657-bib-0031] Hughes, A. C. , M. C. Orr , K. Ma , et al. 2021. “Sampling Biases Shape Our View of the Natural World.” Ecography 44: 1259–1269.

[ece370657-bib-0032] Jackson, A. C. , and J. Mcilvenny . 2011. “Coastal Squeeze on Rocky Shores in Northern Scotland and Some Possible Ecological Impacts.” Journal of Experimental Marine Biology and Ecology 400: 314–321.

[ece370657-bib-0033] Kaplanis, N. J. , C. B. Edwards , Y. Eynaud , and J. E. Smith . 2020. “Future Sea‐Level Rise Drives Rocky Intertidal Habitat Loss and Benthic Community Change.” PeerJ 8: e9186. 32523810 10.7717/peerj.9186 PMC7263295

[ece370657-bib-0034] Kingsbury, K. M. , B. M. Gillanders , D. J. Booth , and I. Nagelkerken . 2020. “Trophic Niche Segregation Allows Range‐Extending Coral Reef Fishes to Co‐Exist With Temperate Species Under Climate Change.” Global Change Biology 26: 721–733. 31846164 10.1111/gcb.14898

[ece370657-bib-0035] Kreft, H. , and W. Jetz . 2010. “A Framework for Delineating Biogeographical Regions Based on Species Distributions.” Journal of Biogeography 37: 2029–2053.

[ece370657-bib-0036] Laliberté, E. , and P. Legendre . 2010. “A Distance‐Based Framework for Measuring Functional Diversity From Multiple Traits.” Ecology 91: 299–305. 20380219 10.1890/08-2244.1

[ece370657-bib-0037] Lamanna, C. , B. Blonder , C. Violle , et al. 2014. “Functional Trait Space and the Latitudinal Diversity Gradient.” Proceedings of the National Academy of Sciences 111: 13745–13750. 10.1073/pnas.1317722111 PMC4183280 25225365

[ece370657-bib-0038] Laureto, L. M. O. , M. V. Cianciaruso , and D. S. M. Samia . 2015. “Functional Diversity: An Overview of Its History and Applicability.” Natureza & Conservação 13: 112–116.

[ece370657-bib-0039] Leitão, R. P. , J. Zuanon , S. Villéger , et al. 2016. “Rare Species Contribute Disproportionately to the Functional Structure of Species Assemblages.” Proceedings of the Royal Society B: Biological Sciences 283: 20160084. 10.1098/rspb.2016.0084 PMC4843652 27053754

[ece370657-bib-0040] Lowe, R. J. , M. V. W. Cuttler , and J. E. Hansen . 2021. “Climatic Drivers of Extreme Sea Level Events Along the Coastline of Western Australia.” Earth's Future 9: e2020EF001620.

[ece370657-bib-0041] Madin, E. M. P. , N. C. Ban , Z. A. Doubleday , T. H. Holmes , G. T. Pecl , and F. Smith . 2012. “Socio‐Economic and Management Implications of Range‐Shifting Species in Marine Systems.” Global Environmental Change 22: 137–146.

[ece370657-bib-0042] Magneville, C. , N. Loiseau , C. Albouy , et al. 2022. “mFD: An R Package to Compute and Illustrate the Multiple Facets of Functional Diversity.” Ecography 2022: 1–15.

[ece370657-bib-0043] Martins, G. M. , F. Arenas , A. I. Neto , and S. R. Jenkins . 2012. “Effects of Fishing and Regional Species Pool on the Functional Diversity of Fish Communities.” PLoS One 7: e44297. 22952950 10.1371/journal.pone.0044297 PMC3432072

[ece370657-bib-0044] Marzloff, M. P. , E. C. J. Oliver , N. S. Barrett , et al. 2018. “Differential Vulnerability to Climate Change Yields Novel Deep‐Reef Communities.” Nature Climate Change 8: 873–878.

[ece370657-bib-0045] McGill, B. J. , M. Dornelas , N. J. Gotelli , and A. E. Magurran . 2015. “Fifteen Forms of Biodiversity Trend in the Anthropocene.” Trends in Ecology & Evolution 30: 104–113. 25542312 10.1016/j.tree.2014.11.006

[ece370657-bib-0046] McLean, M. , D. Mouillot , M. Lindegren , et al. 2019. “Fish Communities Diverge in Species but Converge in Traits Over Three Decades of Warming.” Global Change Biology 25: 3972–3984. 31376310 10.1111/gcb.14785

[ece370657-bib-0047] McWilliam, M. , M. O. Hoogenboom , A. H. Baird , C.‐Y. Kuo , J. S. Madin , and T. P. Hughes . 2018. “Biogeographical Disparity in the Functional Diversity and Redundancy of Corals.” Proceedings of the National Academy of Sciences 115: 3084–3089. 10.1073/pnas.1716643115 PMC5866567 29507193

[ece370657-bib-0048] McWilliam, M. , M. S. Pratchett , M. O. Hoogenboom , and T. P. Hughes . 2020. “Deficits in Functional Trait Diversity Following Recovery on Coral Reefs.” Proceedings of the Royal Society B: Biological Sciences 287: 20192628. 10.1098/rspb.2019.2628 PMC7003456 31910784

[ece370657-bib-0049] Micheli, F. , and B. S. Halpern . 2005. “Low Functional Redundancy in Coastal Marine Assemblages.” Ecology Letters 8: 391–400.

[ece370657-bib-0050] Miller, M. G. R. , J. D. Reimer , B. Sommer , et al. 2023. “Temperate Functional Niche Availability Not Resident‐Invader Competition Shapes Tropicalisation in Reef Fishes.” Nature Communications 14: 2181. 10.1038/s41467-023-37550-5 PMC10110547 37069145

[ece370657-bib-0051] Miloslavich, P. , J. J. Cruz‐Motta , E. Klein , et al. 2013. “Large‐Scale Spatial Distribution Patterns of Gastropod Assemblages in Rocky Shores.” PLoS One 8: e71396. 23967204 10.1371/journal.pone.0071396 PMC3742765

[ece370657-bib-0052] Miranda, J. R. , D. Mouillot , D. F. Hernandez , A. S. Lopez , T. D. Chi , and L. A. Perez . 2005. “Changes in Four Complementary Facets of Fish Diversity in a Tropical Coastal Lagoon After 18 Years: A Functional Interpretation.” Marine Ecology. Progress Series (Halstenbek) 304: 1–13.

[ece370657-bib-0053] Monaco, C. J. , C. J. A. Bradshaw , D. J. Booth , B. M. Gillanders , D. S. Schoeman , and I. Nagelkerken . 2020. “Dietary Generalism Accelerates Arrival and Persistence of Coral‐Reef Fishes in Their Novel Ranges Under Climate Change.” Global Change Biology 26: 5564–5573. 32530107 10.1111/gcb.15221

[ece370657-bib-0054] Mora, C. , O. Aburto‐Oropeza , A. Ayala Bocos , et al. 2011. “Global Human Footprint on the Linkage Between Biodiversity and Ecosystem Functioning in Reef Fishes.” PLoS Biology 9: e1000606. 21483714 10.1371/journal.pbio.1000606 PMC3071368

[ece370657-bib-0055] Mouillot, D. , D. R. Bellwood , C. Baraloto , et al. 2013a. “Rare Species Support Vulnerable Functions in High‐Diversity Ecosystems.” PLoS Biology 11: e1001569. 23723735 10.1371/journal.pbio.1001569 PMC3665844

[ece370657-bib-0056] Mouillot, D. , N. A. J. Graham , S. Villéger , N. W. H. Mason , and D. R. Bellwood . 2013b. “A Functional Approach Reveals Community Responses to Disturbances.” Trends in Ecology & Evolution 28: 167–177. 23141923 10.1016/j.tree.2012.10.004

[ece370657-bib-0057] Mouillot, D. , S. Villéger , V. Parravicini , et al. 2014. “Functional Over‐Redundancy and High Functional Vulnerability in Global Fish Faunas on Tropical Reefs.” Proceedings of the National Academy of Sciences of the United States of America 111: 13757–13762. 25225388 10.1073/pnas.1317625111 PMC4183327

[ece370657-bib-0058] Mouton, T. L. , J. D. Tonkin , F. Stephenson , P. Verburg , and M. Floury . 2020. “Increasing Climate‐Driven Taxonomic Homogenization but Functional Differentiation Among River Macroinvertebrate Assemblages.” Global Change Biology 26: 6904–6915. 33030282 10.1111/gcb.15389

[ece370657-bib-0059] Murley, M. , R. Hovey , and J. Prince . 2024. “Biogeography of Invertebrates on Intertidal Rock Platforms in Western Australia, Unpublished.” The University of Western Australia.

[ece370657-bib-0060] Myers, E. M. V. , M. J. Anderson , L. Liggins , E. S. Harvey , C. D. Roberts , and D. Eme . 2021a. “High Functional Diversity in Deep‐Sea Fish Communities and Increasing Intraspecific Trait Variation With Increasing Latitude.” Ecology and Evolution 11: 10600–10612. 34367600 10.1002/ece3.7871 PMC8328419

[ece370657-bib-0061] Myers, E. M. V. , D. Eme , L. Liggins , E. S. Harvey , C. D. Roberts , and M. J. Anderson . 2021b. “Functional Beta Diversity of New Zealand Fishes: Characterising Morphological Turnover Along Depth and Latitude Gradients, With Derivation of Functional Bioregions.” Austral Ecology 46: 965–981.

[ece370657-bib-0062] Oliver, T. H. , M. S. Heard , N. J. B. Isaac , et al. 2015. “Biodiversity and Resilience of Ecosystem Functions.” Trends in Ecology & Evolution (Amsterdam) 30: 673–684. 10.1016/j.tree.2015.08.009 26437633

[ece370657-bib-0063] Paine, R. 1994. Marine Rocky Shores and Community Ecology: An Experimentalist's Perspective. Germany: Ecology Institute.

[ece370657-bib-0064] Pinto‐Ledezma, J. N. , D. J. Larkin , and J. Cavender‐Bares . 2018. “Patterns of Beta Diversity of Vascular Plants and Their Correspondence With Biome Boundaries Across North America.” Frontiers in Ecology and Evolution 6: 1–13.

[ece370657-bib-0065] Przeslawski, R. , S. Ahyong , M. Byrne , G. Wörheide , and P. A. T. Hutchings . 2008. “Beyond Corals and Fish: The Effects of Climate Change on Noncoral Benthic Invertebrates of Tropical Reefs.” Global Change Biology 14: 2773–2795.

[ece370657-bib-0066] QGIS Development Team . 2023. QGIS Geographic Information System (Version 3.24.1). Bern, Switzerland: Open Source Geospatial Foundation Project.

[ece370657-bib-0067] R Core Team . 2022. R: A Language and Environment for Statistical Computing. Vienna, Austria: R Foundation for Statistical Computing.

[ece370657-bib-0068] Ramírez‐Ortiz, G. , L. E. Calderon‐Aguilera , H. Reyes‐Bonilla , et al. 2017. “Functional Diversity of Fish and Invertebrates in Coral and Rocky Reefs of the Eastern Tropical Pacific.” Marine Ecology (Berlin, West) 38: e12447.

[ece370657-bib-0069] Rojas‐Montiel, B. , H. Reyes‐Bonilla , L. E. Calderon‐Aguilera , et al. 2020. “Echinoderm Functional Diversity Does Not Correlate With the Protection Level of Marine Protected Areas in the Mexican Pacific.” Biodiversity and Conservation 29: 1871–1896.

[ece370657-bib-0070] Roxy, M. K. , C. Gnanaseelan , A. Parekh , et al. 2020. Indian Ocean Warming. Singapore: Springer.

[ece370657-bib-0071] Rullens, V. , S. Mangan , F. Stephenson , et al. 2022. “Understanding the Consequences of Sea Level Rise: The Ecological Implications of Losing Intertidal Habitat.” New Zealand Journal of Marine and Freshwater Research 56: 353–370.

[ece370657-bib-0072] Rumm, A. , F. Foeckler , F. Dziock , et al. 2018. “Shifts in Mollusc Traits Following Floodplain Reconnection: Testing the Response of Functional Diversity Components.” Freshwater Biology 63: 505–517.

[ece370657-bib-0073] Sahin, D. E. , N. Bosch , C. Cooper , et al. 2024. “Spatial Structuring of Coral Traits Along a Subtropical‐Temperate Transition Zone Persists Despite Localised Signs of Tropicalisation.” Coral Reefs 43: 1659–1671.

[ece370657-bib-0074] Schaefer, N. , M. Mayer‐Pinto , K. J. Griffin , E. L. Johnston , W. Glamore , and K. A. Dafforn . 2020. “Predicting the Impact of Sea‐Level Rise on Intertidal Rocky Shores With Remote Sensing.” Journal of Environmental Management 261: 110203. 32148273 10.1016/j.jenvman.2020.110203

[ece370657-bib-0075] Schumm, M. , S. M. Edie , K. S. Collins , et al. 2019. “Common Latitudinal Gradients in Functional Richness and Functional Evenness Across Marine and Terrestrial Systems.” Proceedings of the Royal Society B: Biological Sciences 286: 20190745. 10.1098/rspb.2019.0745 PMC6710603 31362632

[ece370657-bib-0076] Scrosati, R. A. , and J. A. Ellrich . 2024. “Changes in the Composition of Invertebrate Assemblages From Wave‐Exposed Intertidal Mussel Stands Along the Nova Scotia Coast, Canada.” PeerJ 12: e17697. 38993978 10.7717/peerj.17697 PMC11238722

[ece370657-bib-0077] Siefert, A. , C. Ravenscroft , M. D. Weiser , and N. G. Swenson . 2013. “Functional Beta‐Diversity Patterns Reveal Deterministic Community Assembly Processes in Eastern North American Trees.” Global Ecology and Biogeography 22: 682–691.

[ece370657-bib-0078] Sigovini, M. , E. Keppel , and D. Tagliapietra . 2016. “Open Nomenclature in the Biodiversity Era.” Methods in Ecology and Evolution 7: 1217–1225.

[ece370657-bib-0079] Smith, H. L. , M. J. Anderson , B. M. Gillanders , S. D. Connell , and M. Dawson . 2014. “Longitudinal Variation and Effects of Habitat on Biodiversity of Australasian Temperate Reef Fishes.” Journal of Biogeography 41: 2128–2139.

[ece370657-bib-0080] Somerfield, P. , K. Clarke , R. Warwick , and N. Dulvy . 2008. “Average Functional Distinctness as a Measure of the Composition of Assemblages.” ICES Journal of Marine Science 65: 1462–1468.

[ece370657-bib-0081] Stuart‐Smith, R. D. , A. E. Bates , J. S. Lefcheck , et al. 2013. “Integrating Abundance and Functional Traits Reveals New Global Hotspots of Fish Diversity.” Nature (London) 501: 539–542. 24067714 10.1038/nature12529

[ece370657-bib-0082] Swenson, N. G. , P. Anglada‐Cordero , and J. A. Barone . 2011. “Deterministic Tropical Tree Community Turnover: Evidence From Patterns of Functional Beta Diversity Along an Elevational Gradient.” Proceedings of the Royal Society B: Biological Sciences 278: 877–884. 10.1098/rspb.2010.1369 PMC3049044 20861048

[ece370657-bib-0083] Swenson, N. G. , and M. D. Weiser . 2014. “On the Packing and Filling of Functional Space in Eastern North American Tree Assemblages.” Ecography 37: 1056–1062.

[ece370657-bib-0084] Thorner, J. , L. Kumar , and S. D. A. Smith . 2014. “Impacts of Climate‐Change‐Driven Sea Level Rise on Intertidal Rocky Reef Habitats Will be Variable and Site Specific.” PLoS One 9: e86130. 24465915 10.1371/journal.pone.0086130 PMC3899206

[ece370657-bib-0085] Thyrring, J. , and L. S. Peck . 2021. “Global Gradients in Intertidal Species Richness and Functional Groups.” eLife 10: 1–17. 10.7554/eLife.64541 PMC8032391 33739285

[ece370657-bib-0086] Tuckett, C. A. , T. de Bettignies , J. Fromont , and T. Wernberg . 2017. “Expansion of Corals on Temperate Reefs: Direct and Indirect Effects of Marine Heatwaves.” Coral Reefs 36: 947–956.

[ece370657-bib-0087] Underwood, A. J. 2000. “Experimental Ecology of Rocky Intertidal Habitats: What Are We Learning?” Journal of Experimental Marine Biology and Ecology 250: 51–76. 10969163 10.1016/s0022-0981(00)00179-9

[ece370657-bib-0088] Vergés, A. , E. Mccosker , M. Mayer‐Pinto , et al. 2019. “Tropicalisation of Temperate Reefs: Implications for Ecosystem Functions and Management Actions.” Functional Ecology 33: 1000–1013.

[ece370657-bib-0089] Villéger, S. , G. Grenouillet , and S. Brosse . 2013. “Decomposing Functional β‐Diversity Reveals That Low Functional β‐Diversity Is Driven by Low Functional Turnover in European Fish Assemblages.” Global Ecology and Biogeography 22: 671–681.

[ece370657-bib-0090] Villéger, S. , N. W. H. Mason , and D. Mouillot . 2008. “New Multidimensional Functional Diversity Indices for a Multifaceted Framework in Functional Ecology.” Ecology 89: 2290–2301. 18724739 10.1890/07-1206.1

[ece370657-bib-0091] Vinagre, C. , V. Mendonça , A. A. V. Flores , A. Baeta , and J. C. Marques . 2018. “Complex Food Webs of Tropical Intertidal Rocky Shores (SE Brazil) – An Isotopic Perspective.” Ecological Indicators 95: 485–491.

[ece370657-bib-0092] Violle, C. , M.‐L. Navas , D. Vile , et al. 2007. “Let the Concept of Trait Be Functional!” Oikos 116: 882–892.

[ece370657-bib-0093] Violle, C. , W. Thuiller , N. Mouquet , et al. 2017. “Functional Rarity: The Ecology of Outliers.” Trends in Ecology & Evolution 32: 356–367. 28389103 10.1016/j.tree.2017.02.002 PMC5489079

[ece370657-bib-0094] Walker, B. 1995. “Conserving Biological Diversity Through Ecosystem Resilience.” Conservation Biology 9: 747–752.

[ece370657-bib-0107] Weiher, E. , G. D. P. Clarke , and P. A. Keddy . 1998. “Community Assembly Rules, Morphological Dispersion, and the Coexistence of Plant Species.” Oikos 81, no. 2: 309. 10.2307/3547051.

[ece370657-bib-0095] Wells, F. E. 1980. “The Distribution of Shallow‐Water Marine Prosobranch Gastropod Molluscs Along the Coast Line of Western Australia.” Veliger 223: 232–247.

[ece370657-bib-0096] Wernberg, T. , S. Bennett , R. C. Babcock , et al. 2016. “Climate‐Driven Regime Shift of a Temperate Marine Ecosystem.” Science (American Association for the Advancement of Science) 353: 169–172. 10.1126/science.aad8745 27387951

[ece370657-bib-0097] Whittaker, R. H. 1972. “Evolution and Measurement of Species Diversity.” Taxon 21: 213–251.

[ece370657-bib-0098] Wilson, B. R. 1993a. Australian Marine Shells Part One: Prosobranch Gastropods. Leederville, W.A: Odyssey Publishing.

[ece370657-bib-0099] Wilson, B. R. 1993b. Australian Marine Shells Part Two: Neogastropods. Leederville, W.A: Odyssey Publishing.

[ece370657-bib-0100] Wilson, N. G. , and L. A. Kirkendale . 2016. “Putting the ‘Indo’ Back Into the Indo‐Pacific: Resolving Marine Phylogeographic Gaps.” Invertebrate Systematics 30: 86–94.

[ece370657-bib-0101] Woolley, S. N. C. , S. D. Foster , N. J. Bax , et al. 2019. “Bioregions in Marine Environments: Combining Biological and Environmental Data for Management and Scientific Understanding.” Bioscience 70: 48–59.

[ece370657-bib-0102] Worm, B. , E. B. Barbier , N. Beaumont , et al. 2006. “Impacts of Biodiversity Loss on Ocean Ecosystem Services.” Science (American Association for the Advancement of Science) 314: 787–790. 10.1126/science.1132294 17082450

[ece370657-bib-0103] Worm, B. , H. K. Lotze , and T. M. Letcher . 2021. “Chapter 21 – Marine Biodiversity and Climate Change.” In Climate Change (Third Edition), edited by T. Letcher , 445–464. Amsterdam, Netherlands: Elsevier.

[ece370657-bib-0104] Wright, J. P. , S. Naeem , A. Hector , et al. 2006. “Conventional Functional Classification Schemes Underestimate the Relationship With Ecosystem Functioning.” Ecology Letters 9: 111–120. 16958875 10.1111/j.1461-0248.2005.00850.x

[ece370657-bib-0105] Zintzen, V. , M. J. Anderson , C. D. Roberts , and C. E. Diebel . 2011. “Increasing Variation in Taxonomic Distinctness Reveals Clusters of Specialists in the Deep Sea.” Ecography (Copenhagen) 34: 306–317.

[ece370657-bib-0106] Zintzen, V. , M. J. Anderson , C. D. Roberts , E. S. Harvey , and A. L. Stewart . 2017. “Effects of Latitude and Depth on the Beta Diversity of New Zealand Fish Communities.” Scientific Reports 7: 8081. 28808296 10.1038/s41598-017-08427-7 PMC5556088

