# Supplementary material for: Temperate Intertidal Ecosystems are Functionally Richer but More Vulnerable to Loss Than Tropical Ecosystems
Source: Ecol Evol. 2024 Dec 5;14(12):e70657. doi: 10.1002/ece3.70657 (PMC11621237; doi:10.1002/ece3.70657)
Supplement: Supplementary file 1 — Data S1. [file ECE3-14-e70657-s001.docx]

## Supplementary materials

| Table S1: Geographic coordinates and assigned latitudinal level of all intertidal rock platforms surveyed along Western Australian coast. | | | | |
| --- | --- | --- | --- | --- |
| Region | Site | Latitude (°S) | Longitude (°E) | Latitudinal level |
| Southwest | Hamelin Bay | -34.2209 | 115.0147 | 34 |
| Southwest | Foul Bay | -34.2355 | 115.032 | 34 |
| Southwest | Gnarabup | -33.9926 | 114.9887 | 33 |
| Southwest | Ellensbrook | -33.9926 | 114.9876 | 33 |
| Southwest | Yallingup | -33.6393 | 115.024 | 33 |
| Perth metropolitan area | Point Peron North | -32.2654 | 115.6857 | 32 |
| Perth metropolitan area | Cottesloe | -32.0003 | 115.7513 | 32 |
| Perth metropolitan area | Ocean reef | -31.7492 | 115.7245 | 31 |
| Perth metropolitan area | Watermans | -31.8508 | 115.7506 | 31 |
| Perth metropolitan area | Illuka | -31.7416 | 115.7223 | 31 |
| Turquoise coast | Greenhead | -30.0748 | 114.9695 | 30 |
| Turquoise coast | Point Louise | -30.0505 | 114.9552 | 30 |
| Turquoise coast | Hangover Bay | -30.6002 | 115.1011 | 30 |
| Turquoise coast | Port Denison North | -29.2619 | 114.9204 | 29 |
| Turquoise coast | Port Denison south | -29.276 | 114.9127 | 29 |
| Turquoise coast | Drummond Cove | -28.6841 | 114.6051 | 28 |
| Turquoise coast | Coronation beach | -28.5551 | 114.5625 | 28 |
| Turquoise coast | Horrocks | -28.3504 | 114.4066 | 28 |
| Turquoise coast | Siphon road | -27.7215 | 114.1532 | 27 |
| Ningaloo | Gnaraloo Bay In | -23.7721 | 113.5361 | 24 |
| Ningaloo | Gnaraloo Bay out | -23.7636 | 113.5577 | 24 |
| Ningaloo | Three Mile out | -23.8746 | 113.4956 | 24 |
| Ningaloo | Bateman Bay In | -23.0573 | 113.8203 | 23 |
| Ningaloo | Coral Bay north | -23.1531 | 113.7683 | 23 |
| Ningaloo | Coral Bay south | -23.1568 | 113.7665 | 23 |
| Ningaloo | Pilgramunna | -22.194 | 113.855 | 22 |
| Ningaloo | Mandu South | -22.1462 | 113.8696 | 22 |
| Ningaloo | Yardie Creek | -22.3266 | 113.8094 | 22 |
| Ningaloo | Mangrove Bay | -21.961 | 113.9409 | 21 |
| Ningaloo | Jurabi In 1 | -21.8503 | 114.0214 | 21 |
| Ningaloo | Surfers South | -21.7907 | 114.1533 | 21 |
| Pilbara/Kimberley | Finucane Island | -20.2975 | 118.5725 | 20 |
| Pilbara/Kimberley | Cemetery Beach | -20.3058 | 118.6121 | 20 |
| Pilbara/Kimberley | Rock of Ages | -20.2992 | 118.6409 | 20 |
| Pilbara/Kimberley | Cape Keraudren 1 | -19.9587 | 119.773 | 19 |
| Pilbara/Kimberley | Cape Keraudren 2 | -19.9626 | 119.7803 | 19 |
| Pilbara/Kimberley | Shellplace | -18.5796 | 121.7346 | 18 |
| Pilbara/Kimberley | Cowrie | -18.5332 | 121.7736 | 18 |
| Pilbara/Kimberley | Lighthouse | -18.6985 | 121.6271 | 18 |

| Table S2: Functional traits and categories used for gastropod trait matrix construction. Traits based on those used by Floyd et al 2020. | | |
| --- | --- | --- |
| Trait | Categories | Relevance |
| Feeding | Coded using four categories- predators, grazers, deposit feeder, suspension feeder | This trait categorises a taxa's trophic level to represent the varied diets of gastropods. Feeding methods can indicate the availability of food resources within a habitat and distinctions between temperate and tropical gastropod species has been observed in other systems (Valentine, Roy & Jablonski 2002). |
| Position | Coded using three categories – burrowing, attached, free living | This trait categorises a taxa's morphological adaptations to habitat usage and physical positioning on intertidal rock platforms. An organism’s position on rock platforms links to the role a taxa likely plays in a community in terms of habitat modification and its vulnerability to disturbance specific stressors like predation, habitat degradation, fishing pressures etc. |
| Mobility | Coded using two categories – mobile, sessile | This trait captures a taxa’s ability to move through the environment, their role in a carbon and energy transmission and habitat formation (Hyndes et al. 2014). |
| Shape | Coded using seven categories – conic, dorsoventrally compressed, laterally compressed, fusiform, ovate, turbinate, vermiform | This trait categorises the shell/bodyshape morphology of the taxa, which can reflect ability to withstand predation and resistance to physical disturbances e.g. wave or tidal action. |
| Reproduction connectivity | Coded using three categories – direct development, spawning, brooder | This trait reflects the dispersal capabilities of the taxa and captures aspects of life history (Blanco et al. 2019). |
| Max length | Coded using six categories- <5cm, 5-10cm, 1-20cm,20-30cm, 30-40cm, 50cm+ | This trait commonly is used to approximate biomass and is measured from posterior to anterior. Size has been linked to a taxa’s response to environmental disturbances (Feary et al. 2014). |

| Table S3: Eigenvalues for each axis of trait space PCoA and the proportion of variance explained by each axis (Relative_eig) | | |
| --- | --- | --- |
| PCoA | Eigenvalues | Relative_eig |
| 1 | 8.667542 | 0.463138 |
| 2 | 4.494488 | 0.240156 |
| 3 | 3.293942 | 0.176007 |
| 4 | 1.804683 | 0.096431 |
| 5 | 1.573907 | 0.084099 |
| 6 | 1.027246 | 0.054889 |
| 7 | 0.878477 | 0.04694 |
| 8 | 0.725056 | 0.038742 |
| 9 | 0.685809 | 0.036645 |
| 10 | 0.439468 | 0.023482 |

Figure S1: Functional richness (Fric) of gamma intertidal gastropod assemblages across a latitudinal gradient in Western Australia.

Figure S2: Mean Pairwise Functional Distances (MPFD) of Western Australian intertidal gastropod assemblages across a latitude gradient. The median is indicated by the horizontal line within each box. The number of sites sampled in each latitude varies: 34°S n=2, 33°S n=3, 32°S n=2, 31°S n=3, 30°S n=3, 29°S n=2, 28°S n=3, 27°S n=1, 24°S n=3, 23°S n=3, 22°S n=3, 21°S n=3, 20°S n=3, 19°S n=2, 18°S n=3.

Figure S3: Mean Pairwise Functional Distance (MPFD) of gamma intertidal gastropod assemblages across a latitudinal gradient in Western Australia.

Figure S4: Functional Nearest Neighbour Distance (FNND) of gamma intertidal gastropod assemblages across a latitudinal gradient in Western Australia.

Figure S5: Beta diversity (Jaccard dissimilarity) for within latitude functional turnover. The number of sites sampled in each latitude varies: 34°S n=2, 33°S n=3, 32°S n=2, 31°S n=3, 30°S n=3, 29°S n=2, 28°S n=3, 27°S n=1, 24°S n=3, 23°S n=3, 22°S n=3, 21°S n=3, 20°S n=3, 19°S n=2, 18°S n=3.

Figure S6: Jaccard dissimilarity value for gamma assemblage turnover plotted against distances between pairs of latitudes.

| **Table S4: Components of site functional beta diversity (jac_diss) based on Jaccard dissimilarities, decomposed into turnover (jac_turn) and nestedness (jac_nest ) components.** | | | | |
| --- | --- | --- | --- | --- |
| Site | **Site** | **jac_diss** | **jac_turn** | **jac_nest** |
| Perth metroN | Kalbarri | 0.92898 | 7.36E-16 | 0.92898 |
| Kalbarri | Ningaloo South2 | 0.92314 | 0 | 0.92314 |
| Kalbarri | Ningaloo North2 | 0.9131 | 0.03311 | 0.87999 |
| Kalbarri | Kimberley | 0.91013 | 0.0417 | 0.86842 |
| Kalbarri | Ningaloo South | 0.91007 | 0 | 0.91007 |
| Southwest | Kalbarri | 0.90949 | 0 | 0.90949 |
| Jurien | Kalbarri | 0.90902 | 0 | 0.90902 |
| Kalbarri | Ningaloo North | 0.90746 | 7.36E-16 | 0.90746 |
| Kalbarri | Port Hedland | 0.89043 | 0.11736 | 0.77307 |
| Perth metro | Kalbarri | 0.8804 | 0.26868 | 0.61171 |
| SouthwestN | Kalbarri | 0.8738 | 0 | 0.8738 |
| Kalbarri | Cape Keraudren | 0.86482 | 7.36E-16 | 0.86482 |
| Geraldton | Kalbarri | 0.84078 | 0 | 0.84078 |
| Port Denison | Kalbarri | 0.7921 | 0.03119 | 0.76091 |
| Southwest | Port Hedland | 0.70772 | 0.64834 | 0.05938 |
| Perth metro | Perth metroN | 0.70217 | 0.46485 | 0.23732 |
| Southwest | Perth metro | 0.67075 | 0.52524 | 0.14551 |
| Perth metro | Cape Keraudren | 0.66963 | 0.65388 | 0.01575 |
| Perth metroN | Port Denison | 0.66581 | 0.00404 | 0.66178 |
| Geraldton | Ningaloo North2 | 0.66112 | 0.45156 | 0.20956 |
| Southwest | Ningaloo North2 | 0.65758 | 0.65238 | 0.0052 |
| Southwest | Cape Keraudren | 0.65532 | 0.53031 | 0.12501 |
| Perth metro | Ningaloo South2 | 0.65426 | 0.4117 | 0.24255 |
| Perth metroN | Port Hedland | 0.65181 | 0.47758 | 0.17423 |
| Perth metro | Kimberley | 0.64994 | 0.49937 | 0.15057 |
| Perth metro | Geraldton | 0.64722 | 0.62296 | 0.02426 |
| Perth metro | Ningaloo North | 0.6431 | 0.49117 | 0.15193 |
| Perth metro | Ningaloo South | 0.64268 | 0.47677 | 0.16591 |
| Port Denison | Ningaloo South2 | 0.63772 | 0.00139 | 0.63634 |
| Perth metroN | Cape Keraudren | 0.63525 | 0.36608 | 0.26917 |
| Geraldton | Port Hedland | 0.62543 | 0.52825 | 0.09718 |
| Ningaloo North2 | Cape Keraudren | 0.61477 | 0.45821 | 0.15657 |
| Port Denison | Kimberley | 0.60619 | 0.12486 | 0.48133 |
| Perth metro | Jurien | 0.60539 | 0.42125 | 0.18414 |
| Port Denison | Cape Keraudren | 0.60497 | 0.4287 | 0.17627 |
| Southwest | Kimberley | 0.60152 | 0.59689 | 0.00463 |
| Perth metroN | Ningaloo North2 | 0.60106 | 0.52879 | 0.07227 |
| Southwest | Ningaloo South2 | 0.59906 | 0.54735 | 0.05172 |
| Southwest | Port Denison | 0.59756 | 0.07885 | 0.51872 |
| Perth metro | Port Hedland | 0.59583 | 0.52747 | 0.06836 |
| SouthwestN | Port Hedland | 0.58706 | 0.5657 | 0.02136 |
| Port Denison | Ningaloo North2 | 0.58677 | 0.01474 | 0.57204 |
| Southwest | Ningaloo South | 0.58185 | 0.57994 | 0.00191 |
| Port Denison | Ningaloo South | 0.57913 | 0.01128 | 0.56785 |
| Jurien | Port Denison | 0.57526 | 0.01456 | 0.5607 |
| Perth metro | Ningaloo North2 | 0.57279 | 0.34866 | 0.22412 |
| Perth metroN | Ningaloo North | 0.57027 | 0.47075 | 0.09953 |
| Port Denison | Ningaloo North | 0.56961 | 0.01966 | 0.54995 |
| SouthwestN | Ningaloo North2 | 0.56884 | 0.42419 | 0.14465 |
| Perth metro | Port Denison | 0.55866 | 0.39415 | 0.16451 |
| Perth metroN | Geraldton | 0.55797 | 0.01255 | 0.54542 |
| Perth metroN | Kimberley | 0.54647 | 0.44209 | 0.10438 |
| Perth metroN | Ningaloo South2 | 0.54301 | 0.51514 | 0.02787 |
| Ningaloo North | Port Hedland | 0.53435 | 0.43523 | 0.09912 |
| Jurien | Cape Keraudren | 0.53241 | 0.34433 | 0.18808 |
| Port Hedland | Cape Keraudren | 0.53229 | 0.47953 | 0.05276 |
| Southwest | Ningaloo North | 0.52778 | 0.51995 | 0.00783 |
| SouthwestN | Perth metro | 0.52558 | 0.47231 | 0.05327 |
| Port Denison | Port Hedland | 0.5253 | 0.17934 | 0.34597 |
| Perth metroN | Ningaloo South | 0.52496 | 0.4254 | 0.09956 |
| SouthwestN | Cape Keraudren | 0.52437 | 0.49897 | 0.02539 |
| Jurien | Ningaloo North | 0.51939 | 0.51326 | 0.00613 |
| Geraldton | Cape Keraudren | 0.51905 | 0.45291 | 0.06615 |
| Geraldton | Ningaloo South2 | 0.51869 | 0.00399 | 0.5147 |
| Ningaloo North2 | Kimberley | 0.51123 | 0.49673 | 0.0145 |
| Southwest | Jurien | 0.50372 | 0.5018 | 0.00192 |
| Ningaloo South | Ningaloo North2 | 0.49677 | 0.49069 | 0.00608 |
| Ningaloo North | Cape Keraudren | 0.49178 | 0.29174 | 0.20004 |
| SouthwestN | Ningaloo South2 | 0.48892 | 0.19237 | 0.29655 |
| Ningaloo North | Ningaloo North2 | 0.48762 | 0.46981 | 0.01782 |
| Jurien | Port Hedland | 0.47805 | 0.35162 | 0.12643 |
| SouthwestN | Perth metroN | 0.47635 | 0.08728 | 0.38907 |
| Geraldton | Kimberley | 0.46771 | 0.09795 | 0.36976 |
| SouthwestN | Kimberley | 0.46208 | 0.29129 | 0.1708 |
| Ningaloo South2 | Cape Keraudren | 0.45504 | 0.05233 | 0.4027 |
| Jurien | Ningaloo North2 | 0.45041 | 0.43848 | 0.01193 |
| Perth metroN | Jurien | 0.44328 | 0.31122 | 0.13206 |
| Southwest | Geraldton | 0.43879 | 0.01623 | 0.42256 |
| Geraldton | Ningaloo South | 0.43728 | 0.00478 | 0.4325 |
| SouthwestN | Ningaloo South | 0.43656 | 0.23613 | 0.20043 |
| Ningaloo South | Port Hedland | 0.43564 | 0.28624 | 0.1494 |
| Jurien | Geraldton | 0.43063 | 0.00453 | 0.4261 |
| Geraldton | Ningaloo North | 0.43034 | 0.02501 | 0.40533 |
| Jurien | Kimberley | 0.42982 | 0.42472 | 0.0051 |
| SouthwestN | Ningaloo North | 0.42908 | 0.24703 | 0.18205 |
| Jurien | Ningaloo South | 0.41552 | 0.41011 | 0.0054 |
| Port Hedland | Kimberley | 0.41155 | 0.26929 | 0.14226 |
| SouthwestN | Port Denison | 0.41145 | 0.01442 | 0.39703 |
| Ningaloo South2 | Ningaloo North2 | 0.40353 | 0.32791 | 0.07562 |
| Ningaloo South2 | Port Hedland | 0.37392 | 0.04934 | 0.32458 |
| Port Denison | Geraldton | 0.37125 | 0.18192 | 0.18933 |
| Southwest | SouthwestN | 0.37039 | 0.13939 | 0.231 |
| Ningaloo South | Cape Keraudren | 0.35358 | 0.03382 | 0.31976 |
| Cape Keraudren | Kimberley | 0.34432 | 0.04334 | 0.30098 |
| Jurien | Ningaloo South2 | 0.32297 | 0.21178 | 0.11119 |
| Ningaloo South2 | Ningaloo North | 0.30402 | 0.17443 | 0.12959 |
| SouthwestN | Jurien | 0.3006 | 0.03444 | 0.26615 |
| Ningaloo North | Kimberley | 0.2846 | 0.28107 | 0.00353 |
| Ningaloo North2 | Port Hedland | 0.27631 | 0.04267 | 0.23364 |
| Ningaloo South | Ningaloo North | 0.26171 | 0.24291 | 0.0188 |
| Southwest | Perth metroN | 0.24636 | 0.04417 | 0.20218 |
| SouthwestN | Geraldton | 0.23342 | 0.03657 | 0.19685 |
| Ningaloo South2 | Kimberley | 0.21949 | 0.07111 | 0.14838 |
| Ningaloo South | Ningaloo South2 | 0.2048 | 0.0746 | 0.13019 |

Figure S7: Number of functional entities per site at each latitude. The median is indicated by the horizontal line within each box. The number of sites sampled in each latitude varies: 34°S n=2, 33°S n=3, 32°S n=2, 31°S n=3, 30°S n=3, 29°S n=2, 28°S n=3, 27°S n=1, 24°S n=3, 23°S n=3, 22°S n=3, 21°S n=3, 20°S n=3, 19°S n=2, 18°S n=3.

Figure S8: The relationship between the number of functional entities and species richness across sites at each latitude sampled.

Figure S9: Functional redundancy of functional entity assemblages among sites at each latitude. The median is indicated by the horizontal line within each box. The number of sites sampled in each latitude varies: 34°S n=2, 33°S n=3, 32°S n=2, 31°S n=3, 30°S n=3, 29°S n=2, 28°S n=3, 27°S n=1, 24°S n=3, 23°S n=3, 22°S n=3, 21°S n=3, 20°S n=3, 19°S n=2, 18°S n=3.

Figure S10: Functional over redundancy of functional entity assemblages among sites at each latitude. The median is indicated by the horizontal line within each box. The number of sites sampled in each latitude varies: 34°S n=2, 33°S n=3, 32°S n=2, 31°S n=3, 30°S n=3, 29°S n=2, 28°S n=3, 27°S n=1, 24°S n=3, 23°S n=3, 22°S n=3, 21°S n=3, 20°S n=3, 19°S n=2, 18°S n=3.

Figure S11: Functional vulnerability of functional entity assemblages among sites at each latitude. The median is indicated by the horizontal line within each box. The number of sites sampled in each latitude varies: 34°S n=2, 33°S n=3, 32°S n=2, 31°S n=3, 30°S n=3, 29°S n=2, 28°S n=3, 27°S n=1, 24°S n=3, 23°S n=3, 22°S n=3, 21°S n=3, 20°S n=3, 19°S n=2, 18°S n=3.

Figure S12: Dendrogram of UPGMA clustering of FE gamma assemblages for each latitude. The number of sites sampled in each latitude varies: 34°S n=2, 33°S n=3, 32°S n=2, 31°S n=3, 30°S n=3, 29°S n=2, 28°S n=3, 27°S n=1, 24°S n=3, 23°S n=3, 22°S n=3, 21°S n=3, 20°S n=3, 19°S n=2, 18°S n=3.

| Table S5: Pairwise PERMANOVA comparing FE assemblage compositions between functional bioregions. | | | |
| --- | --- | --- | --- |
| Groups | dF | T | P |
| Temperate, Kalbarri | 17 | 1.0153 | 0.5273 |
| Temperate, Tropical | 36 | 2.6559 | 0.0001 |
| Kalbarri, Tropical | 19 | 1.3303 | 0.0453 |


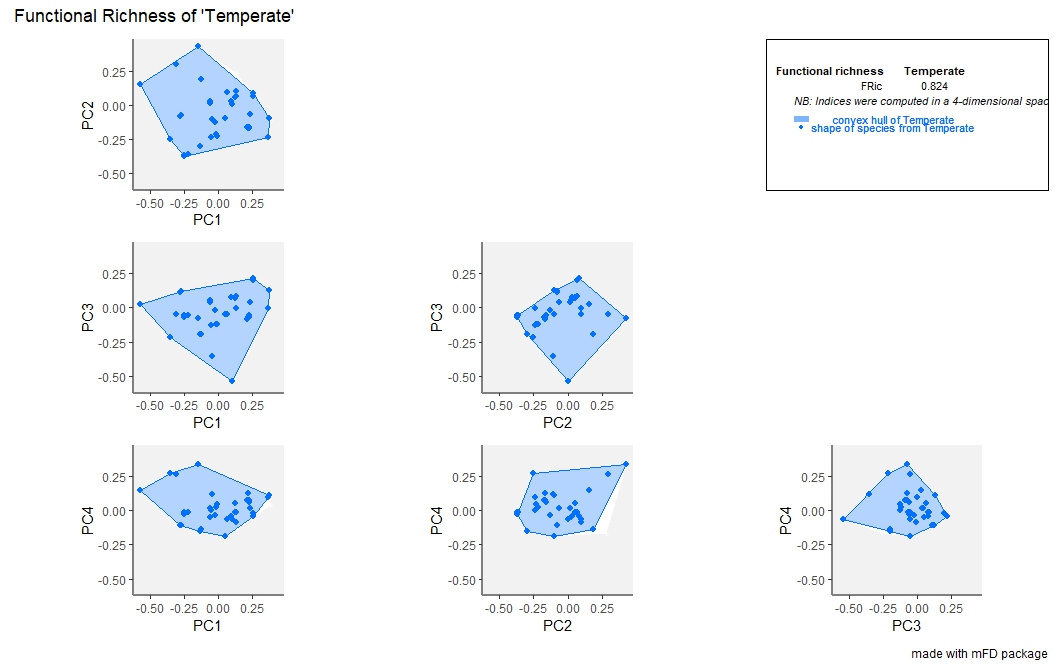

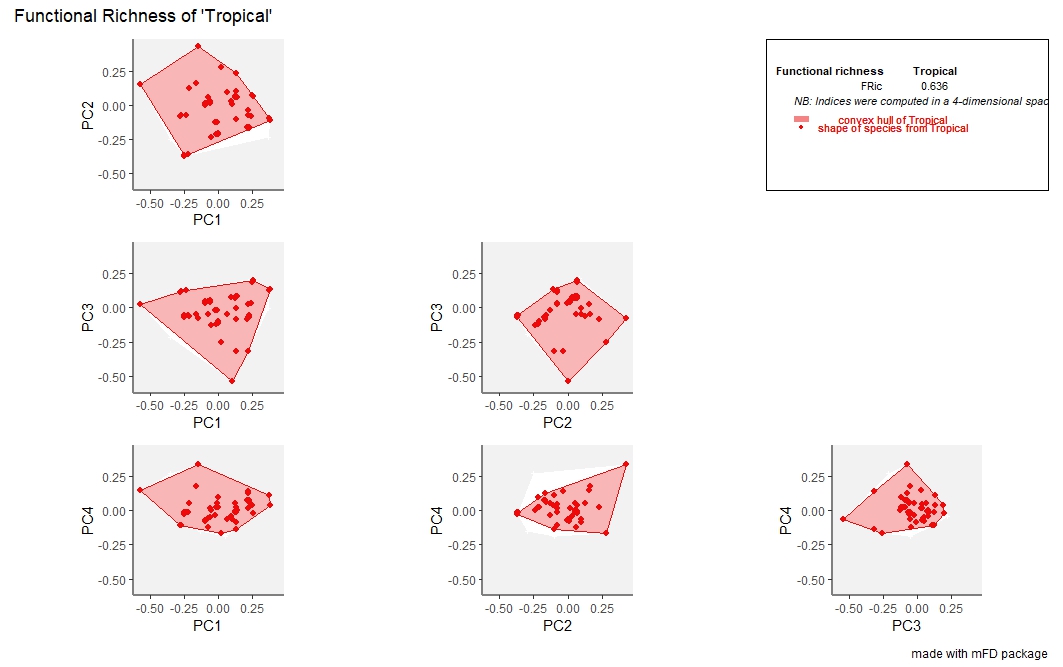


**Figure S13: Functional richness of tropical and temperate bioregions in the global trait space hull. A principal component analysis (PCA) of the global trait space based on the FE latitudinal assemblages revealed with 70% of trait variation expressed could be expressed in two dimensions and 97% in four dimensions. The tropical hull occupied 64% of the global hull and the temperate hull occupied 82%.**
